# Recent Advances in the Synthesis of Glycoconjugates for Vaccine Development

**DOI:** 10.3390/molecules23071712

**Published:** 2018-07-13

**Authors:** Cinzia Colombo, Olimpia Pitirollo, Luigi Lay

**Affiliations:** Dipartimento di Chimica, Universita’ degli Studi di Milano, via Golgi 19, 20133 Milano, Italy; cinzia.colombo@unimi.it (C.C.); olimpia.pitirollo@unimi.it (O.P.)

**Keywords:** bacterial infections, fungal infections, carbohydrates, glycoconjugates, vaccines

## Abstract

During the last decade there has been a growing interest in glycoimmunology, a relatively new research field dealing with the specific interactions of carbohydrates with the immune system. Pathogens’ cell surfaces are covered by a thick layer of oligo- and polysaccharides that are crucial virulence factors, as they mediate receptors binding on host cells for initial adhesion and organism invasion. Since in most cases these saccharide structures are uniquely exposed on the pathogen surface, they represent attractive targets for vaccine design. Polysaccharides isolated from cell walls of microorganisms and chemically conjugated to immunogenic proteins have been used as antigens for vaccine development for a range of infectious diseases. However, several challenges are associated with carbohydrate antigens purified from natural sources, such as their difficult characterization and heterogeneous composition. Consequently, glycoconjugates with chemically well-defined structures, that are able to confer highly reproducible biological properties and a better safety profile, are at the forefront of vaccine development. Following on from our previous review on the subject, in the present account we specifically focus on the most recent advances in the synthesis and preliminary immunological evaluation of next generation glycoconjugate vaccines designed to target bacterial and fungal infections that have been reported in the literature since 2011.

## 1. Introduction

Notwithstanding the great advances of modern medicine, infectious diseases still have a strong impact on public health, both in industrialized and developing countries, due to their significant health-related costs for clinical treatment. In particular, the list of the drug-resistant bacteria is increasing continuously, and novel and more efficient means to prevent microbial infections caused by antibiotic-resistant microorganisms are urgently needed. According to the World Health Organization (WHO) [1], vaccination is the most cost-effective strategy for controlling infections caused by pathogenic microorganisms. Actually, vaccines are able to confer long-term protective immunity on the population and have made possible a great revolution in the 20th century, saving millions of lives.

The surface of bacterial pathogens is covered with a dense array of complex glycans, such as lipopolysaccharide of Gram-negative bacteria and the polysaccharide coat (capsular polysaccharides, CPS) of encapsulated bacteria that are crucial protective antigens and major virulence factors. For example, each strain of *Streptococcus pneumoniae* (the pneumococcus) produces one out of 90 different capsular polysaccharides, which are believed to have been selected as a mechanism to evade the human immune response [2]. All these glycoforms are capable of interacting with the immune system inducing the production of carbohydrate-specific antibodies. They therefore represent attractive targets for vaccine design.

A major drawback of polysaccharide-based vaccines, however, is their limited clinical efficacy. They induce T cell-independent immune responses, featured by poor immunogenicity in children under 5 years of age, in elderly and immunocompromised individuals, and fail to generate conventional B cell-mediated immunological memory. Polysaccharide immunogenicity can be strongly enhanced by conjugation to an immunogenic carrier protein, providing T cell-dependent glycoconjugate antigens able to stimulate B cell maturation to memory cells and induce immunoglobulin class switching from IgM to polysaccharide-specific IgG. The introduction of glycoconjugate vaccines represented one of the keys for success of vaccination, especially for infants and young children who are the most affected population by infectious diseases [3,4,5,6]. Carbohydrate-based antigens needed for inclusion in a glycoconjugate vaccine, however, are not readily available from natural sources. In particular, the isolation and purification of naturally occurring glycans is still a great challenge that may lead to heterogeneous compositions and batch-to-batch variability. A relevant example is the toxic endotoxin lipid A, a major component of the lipopolysaccharide (LPS) of *Shigella flexneri* 2a. The development of LPS-based conjugate vaccines against *Shigella flexneri* requires careful LPS-detoxification, a technically demanding and expensive process which also increases the manufacture costs [7,8]. Hence, the development of cost-effective, glycoconjugate vaccines based on fully synthetic saccharide antigens is gaining growing importance, as demonstrated by the outstanding success of the synthetic vaccine Quimi-Hib [9]. Synthetic glycans, indeed, possess well-defined compositions, affording highly reproducible biological properties and a better safety profile. In addition, synthetic oligosaccharides can help to elucidate the minimal structure of the microbial polysaccharide, referred to as epitope or antigenic determinant [10], that can ensure production of a sufficient amount of bactericidal antibodies to confer long term protective immunity of the host. This step is crucial for the design of a new generation of improved and safer vaccines obtained either from chemical synthesis or bacterial source. Consequently, glycoconjugates based on chemically well-defined oligosaccharide structures are now at the forefront of vaccine development.

**Figure 1:**
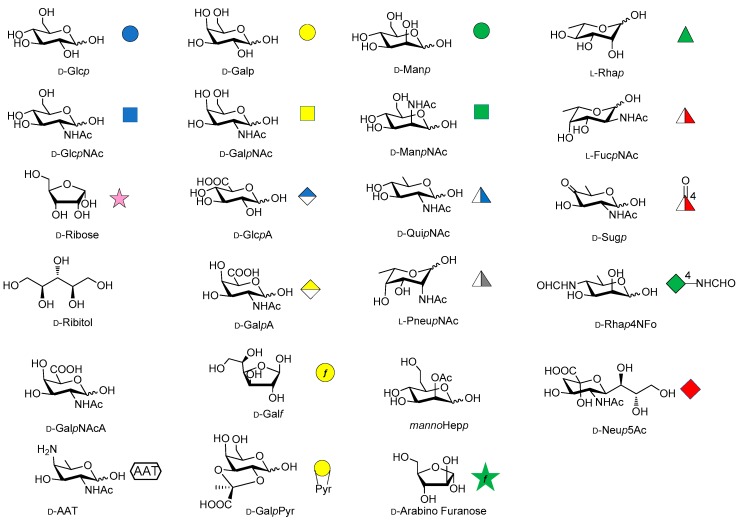
Representation of the monosaccharide building blocks contained in the glycans of pathogen cell wall PS accounted in this manuscript.

Over the last years, the synthesis of complex glycans has made significant progress. A variety of synthetic approaches such as automated solid phase synthesis, one-pot programmable synthesis, enzymatic and improved synthetic methods have introduced new and elegant ways to provide oligosaccharide antigens with well-defined chemical structure for immunological studies. Meanwhile, improved methods for structural elucidation, based on X-ray crystallography, NMR, or in silico studies, as well as advanced techniques to study carbohydrate-protein interactions (glycoarray, surface plasmon resonance, isothermal titration calorimetry, competitive ELISA assay) have been extensively applied to predict the minimal structural requirements needed for the immunological activity of the oligosaccharides. Accordingly, a variety of saccharide fragments reproducing or mimicking the surface carbohydrates of pathogens have been synthesized, coupled to carrier proteins or T cell peptides, and tested for their ability to elicit protective antibodies in animal models. In this regard, in the present review we focus on the most significant advances in the synthesis and preliminary immunological evaluation of synthetic antibacterial and antifungal glycoconjugate vaccine candidates, appeared in the literature from 2011 onwards, following our previous account on the subject [11]. For clarity, the diagrammatic representations of the monosaccharide residues [12] illustrated in Figure 1 above are used throughout this review.

## 2. *Shigella*

The *Shigella* family includes four different groups of Gram-negative bacteria—*S. dysenteriae*, *S. sonnei*, *S. flexneri* and *S. boydii*—each of them comprising different serotypes. *Shigella* is the causative agent of endemic and epidemic shigellosis or bacillary dysentery, an invasive disease of the lower intestine, highly diffused in developing countries and particularly in pediatric population. The development of a fully synthetic glycoconjugate vaccine against *S. dysenteriae* type 1 is currently under investigation, using fragments of the O-antigen of the *Shigella* LPS. Oligomers up to four repeating units of the tetrasaccharide [α-l-Rha-(1→2)-α-d-Gal-(1→3)-α-d-GlcNAc-(1→3)-α-l-Rha] were first synthesized by Pozsgay and covalently linked to human serum albumin (HSA) [13,14]. Preliminary studies showed that the hexadecasaccharide (*n* = 4) is the most immunogenic fragment able to elicit anti O-SP-specific IgG in mice [14] and that the upstream residue (non-reducing end) of the synthetic fragments is crucial for the immunogenicity of these conjugates [15].

**Figure 2:**
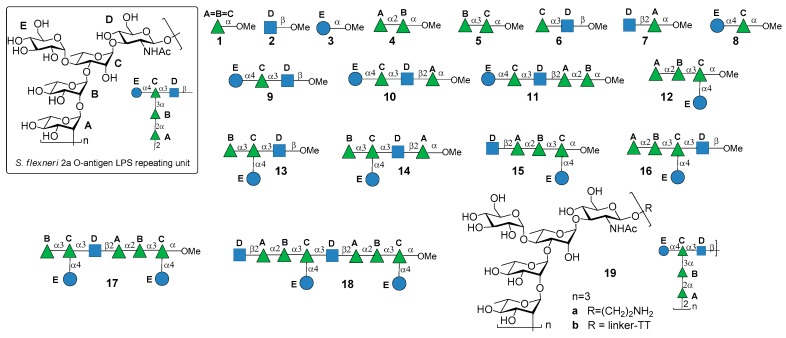
Repeating unit of the *O*-antigen of *S. flexneri* 2a LPS and synthetic fragments reported in [16,17,18].

*S. flexneri* serotype 2a is the most prevalent pathogenic strain in human and a major cause of the endemic form of shigellosis in developing countries. The O-antigen of *S. flexneri* 2a surface LPS is an essential virulence factor and consists of a branched pentasaccharide repeating unit (Figure 2).

In 2005, the Mulard group reported the synthesis of the monomer, dimer and trimer of the pentasaccharide repeating unit (AB(E)CD, Figure 2) and their conjugation to a universal T cell peptide epitope, the pan HLA DR-binding epitope (PADRE) [16,17]. Subsequently, the synthesis of *S. flexneri* serotype 2a O-Ag synthetic fragments **1a**–**18a** (Figure 2) was reported [19]. The antigenicity of all synthetic fragments **1a**–**18a** was evaluated by ELISA assays, in order to identify the immunogenic determinants recognized by five protective mIgGs specific to serotype 2a O-Ag. None of the mono- or disaccharides was recognized, while the sequence ECD (trisaccharide **9a**) was the only one recognized by one mIgG out of five. Tetrasaccharide B(E)CD **13a** was recognized by three of the protective mIgG out of five. The minimal sequences for recognition of all mIgGs were pentasaccharides AB(E)CD **16a** and B(E)CDA **14a**. Following these encouraging results, some selected synthetic oligosaccharides were conjugated to tetanus toxoid (TT) protein and used for immunization studies in mice, leading to the identification of a hit glycoconjugate, **19b**, containing the trimer of the pentasaccharide AB(E)CD. Glycoconjugate **19b** induced an efficient serotype 2a-specific anti-O-Ag Ab response and it was found to be a functional mimic of the native polysaccharide [20]. Recently, Mulard et al. established a reproducible bioconjugation method for the synthesis of the pentadecasaccharide−TT conjugate **19b**, which allowed complete control of the optimal loading [18]. Alum, an adjuvant used in licensed glycoconjugate vaccines like Prevnar 13 or Synflorix, was added to *S. flexneri* serotype 2a vaccine candidate **19b**, which upon immunization was shown to generate a higher and sustained anti-LPS IgG response compared to their nonadjuvanted form [18]. Importantly, Mulard et al. showed that anti-LPS IgG elicited by their synthetic TT conjugate **19b** recognized SF2a bacteria and not only purified SF2a LPS [18]. In addition to these promising findings, the influence of *O*-acetylation of *S. flexneri* 2a O-Ag fragments on antigenicity was studied by Mulard group [21]. Polysaccharide *O*-acetylation has been shown to play a key role for many pathogens in inducing functional Ab responses [22,23,24]. In particular, three diversely *O*-acetylated *S. flexneri* 2a O-Ag decasaccharides were synthesized in homogeneous form and their binding to five different protective mAbs was studied, showing some differences in the recognition patterns. Although these data couldn’t provide an exhaustive proof of the role of *O*-acetylation for *S. flexneri* 2a O-Ag and of the effect of multiple acetates on the antigen, this work showed that studies using synthetic oligosaccharides may contribute to a better understanding of the antigen-antibody molecular recognition event.

## 3. *Clostridium difficile*

*Clostridium difficile* is a Gram-positive, spore-forming anaerobic bacterium causing *Clostridium difficile* infection (CDI), a serious diarrhoeal disease and one of the major cause of hospital-acquired infections (also known as nosocomial infections) in Western countries [25]. The epidemiology of CDI has changed dramatically during this millennium, especially in relation to its clinical presentation, response to treatment and antibiotic resistance [25,26]. In general, after antibiotic treatment that leads to the disruption of the gut microbiota, the intestinal epithelium could be colonized by antibiotic-resistant *C. difficile* spores, which secrete two toxins (toxin A and toxin B) responsible for the clinical symptoms of CDI. Immune-based strategies based on passive administration of monoclonal antibodies against *C. difficile* toxins and surface proteins to treat or prevent CDI in animal models and in clinical trials have been recently reviewed [27,28]. Concurrently, bacterial surface glycans, such as PS-I and PS-II, have been recently proposed as potential target for vaccine development with the aim of preventing bacterial adhesion and colonization.

### 3.1. PS-I-Clostridium difficile

**Figure 3:**
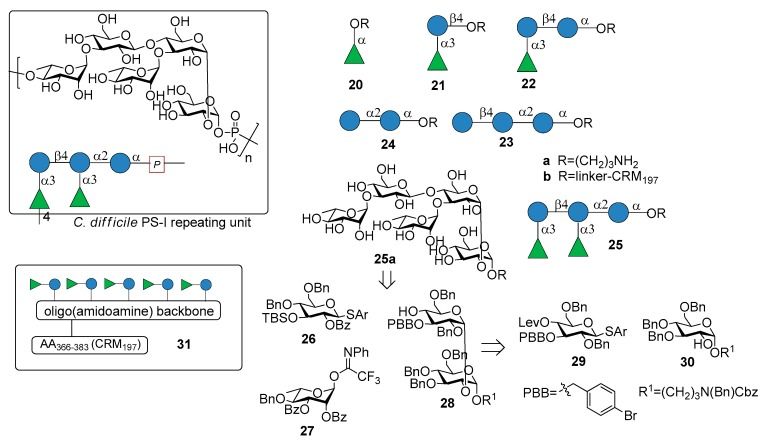
*C. difficile* PS-I repeating unit, synthetic fragments **20**–**25** and retrosynthetic analysis reported in [29].

Recently, Martin et al. reported the synthesis of the pentasaccharide repeating unit of PS-I cell wall polysaccharide of *C. difficile* ribotype 027 (Figure 3), of its related substructures (compounds **20a**–**25a**, Figure 3) and their immunological evaluation for the identification of the minimal epitope [29].

The synthetic fragments were synthesized from monosaccharide building blocks **26**–**30**, linearly proceeding from the downstream end to the upstream end. In particular, the use of the non participating benzyl group at C-2 of thioglycoside **29** provided the condition for 1,2-cis stereoselective glycosylation of glucoside **30**, bearing the linker at the anomeric position. Thioglycoside **29** was functionalized with the orthogonal protecting groups *para*-bromobenzyl (PBB) ether at C-3 and levulinoyl (Lev) ester at C-4 for installation of the branching point.

Glycans **20a**–**25a**, immobilized on microarrays, were screened for antibody recognition with samples from *C. difficile* patients (stools for IgA and serum for IgG). IgA and IgG antibodies specific to all glycan antigens were present in most fecal samples and sera, respectively, of both patients and control groups. Reconvalescent patients showed highly variable antibody levels and statistically higher IgG levels. Pentasaccharide **25a** was conjugated to CRM_197_ (non-toxic mutant of diphtheria toxin) and the resulting glycoconjugate **25b** was injected in mice, inducing Ig class switching, affinity maturation and producing self-specific antibodies, without eliciting antibodies against two control oligosaccharides (*C. difficile* PS-II hexasaccharide and *Leishmania* lipophosphoglycan capping tetrasaccharide). Interestingly, antibodies raised by glycoconjugate **25b** also recognized trisaccharide **22a** and disaccharide α-Rha-(1→3)-Glc **21a**, which was identified as the minimal epitope. Indeed, α-Rha-(1→3)-Glc disaccharide-CRM_197_ conjugate **21b** was able to induce antibodies recognizing the *C. difficile* PS-I pentasaccharide **25a**. In a following work [30], a multivalent presentation of disaccharide **21a** on an oligo(amidoamine) synthetic scaffold [31] was shown to be highly antigenic. In particular, a pentavalent presentation of the disaccharide **31** (Figure 3), built on the oligo(amidoamine) backbone and displaying a T-cell epitope (amino acids 366–383 of the CRM_197_ protein) showed increased antigenicity compared with monovalent **21b**, eliciting antibodies against pentasaccharide **25a**. A detailed investigation of the glycan-antibody binding was conducted with a combination of different techniques like glycan microarray, surface plasmon resonance, interaction map, saturation transfer difference (STD)-NMR and isothermal titration calorimetry (ITC). It was demonstrated that the mAbs mainly interacted with the terminal rhamnose and the adjacent glucose of the disaccharide **21a** and that in pentasaccharide **25a** the linkage connecting the two disaccharides is not directly engaged in antibody binding, although the affinity (K_D_) increases from micromolar for disaccharide **21a** to nanomolar for pentasaccharide **25a**. Both glycoconjugates **21b** and **25b** are currently in preclinical evaluation as novel vaccine candidates against *C. difficile*.

### 3.2. PS-II-Clostridium difficile

**Figure 4:**
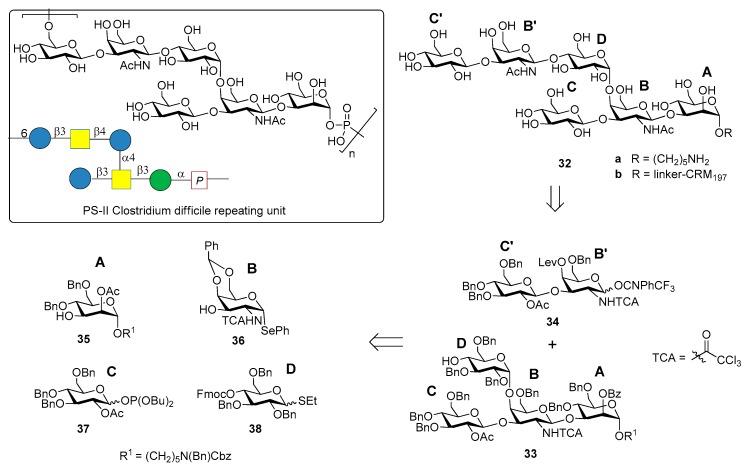
*C. difficile* PS-II repeating unit and retrosynthetic analysis of fragment **32** reported in [32].

The chemical synthesis of the hexasaccharide repeating unit (Figure 4) of PS-II cell wall polysaccharide of *C. difficile* ribotype 027, one of the most virulent strains, with two similar synthetic strategies, was reported simultaneously by two groups in 2011 [32,33].

The synthesis of PS-II oligosaccharide lacking the phosphate group (compound **32a**, Figure 4) was carried out by Oberli et al. [32] via a [4+2] glycosylation of tetrasaccharide AB(D)C **33** with disaccharide B’C’ **34**, starting from monosaccharide building blocks **35**–**38**. Hexasaccharide conjugated to CRM_197_ (**32b**) was used for mice immunization and resulted in the production of IgG antibodies that bound specifically hapten **32a**. In addition, IgA antibodies from the stools of patients diagnosed with CDI (the supernatant stools, and not the serum, were chosen because the contact site with *C. difficile* is the intestinal mucosa) were analyzed. Glycan microarrays containing hexasaccharide antigen **32a** were used to screen patient samples. Anti-PS-II IgA antibodies were found in the stools of patients diagnosed with CDI, suggesting that the synthetic hexasaccharide could be used in a glycoconjugate vaccine candidate against CDI.

**Figure 5:**
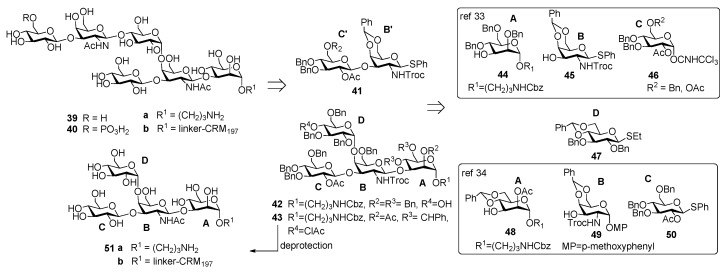
*C. difficile* PSII synthetic fragments reported in [33,34].

Danieli et al. [33] reported the synthesis of hexasaccharide **39a** and its phosphorylated analogue **40a**, starting from disaccharide **41** and tetrasaccharide **42** (or its analogue **43**) via a [4+2] convergent approach (Figure 5).

Tetrasaccharide **42** was in turn prepared from monosaccharide building blocks **44**–**47**, by first assembling the linear trisaccharide ABC and then inserting the α-Glc D unit [33]. An alternative route leading to tetrasaccharide **43** starting from building blocks **48**, **49** and **50** was also developed [34]. Of note, the glycosylation with 4,6-*O*-benzylidene-protected ethylthioglycoside **47** allowed the stereoselective introduction of the 1,2-cis linkage for both routes. Tetrasaccharide AB(D)C **51a**, obtained from deprotection of **43**, was also synthesized to examine the effect of the branching point of the hexaglycosyl unit in determining the immunogenicity. Sera from mice immunized with the PSII-CRM_197_ conjugate were used to check their capability to bind synthetic fragments **39a**, **40a**, **51a**. Tetrasaccharide **51a** showed no binding, while both **39a** and phosphorylated fragment **40a** bound anti-PSII antibodies. In a second experiment, the three synthetic glycans conjugated to CRM_197_ (compounds **39b**, **40b** and **51b**) and native PSII-CRM_197_ were injected in mice and evaluated for their ability to elicit anti PSII antibodies. Sera were analyzed by ELISA for their content of anti PSII IgG, using PSII-HSA for the coating of the plates. Interestingly, only the glycoconjugates obtained from the native polysaccharide and the phosphorylated hexasaccharide **40b** were able to induce IgG antibodies that bound PSII and low levels of anti-PSII IgM antibodies. Tetrasaccharide **51b** and the nonphosphorylated hexasaccharide **39b** elicited self-specific antibodies but did not induce IgG nor IgM anti-PSII titers. A comparison between the studies of Oberli et al. [32] and Adamo et al. [34] reveals that the phosphate group on the hexasaccharide repeating unit of PS-II plays a subtle immunological role. Indeed, the phosphate group is not required to raise IgG antibodies production against hexasaccharide hapten **32a**, while it is a prerequisite to elicit antibodies recognizing, besides the phosphorylated and the nonphosporylated hapten, also the native PS-II polysaccharide. These findings suggested that the charged phosphate is crucial to mimic the native PSII polysaccharide [34] and can be used for the design of carbohydrate antigens as vaccine candidates.

## 4. *Burkholderia pseudomallei*

**Figure 6:**
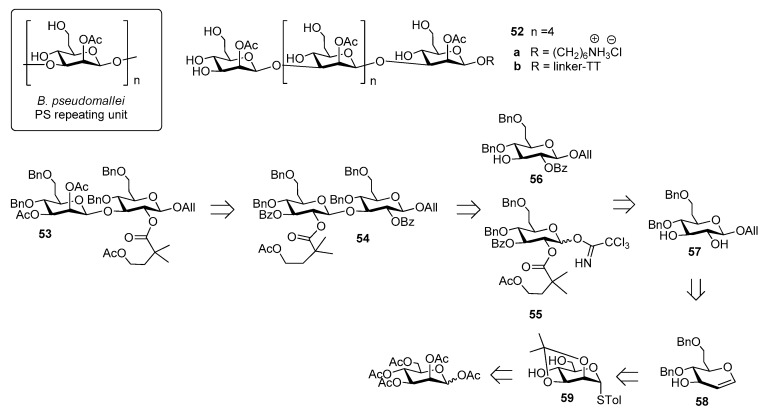
*B. pseudomallei* CPS repeating unit and retrosynthetic analysis of compound **52** reported in [37].

*Burkholderia pseudomallei* is a Gram-negative environmental bacterium which is widespread in the soil and surface water in southeast Asia and northern Australia, causing melioidosis, a serious and often fatal disease presenting acute pulmonary infections, fulminant sepsis and chronic infection mimicking tuberculosis [35]. Antibiotic treatment is usually divided into two phases: a first phase to prevent death from sepsis and a second phase with the aim of preventing recurrence [36]. This protracted treatment is not always successful and mortality rate remains high (from 15% in Australia to 40% in Thailand, approaching 90% with septicaemia) [37]. For this reason, substantial effort has been undertaken to develop vaccine candidates which would protect humans against *B. pseudomallei* infections [38]. Among the identified virulence factors [39], the capsular polysaccharide of *B. pseudomallei*, a homopolymer of 2-*O*-acetyl manno-heptopyranose (Figure 6) has been recently considered for the development of an effective melioidosis vaccine [40]. 

Of note, *B. pseudomallei* CPS is expressed as a unique serotype in all reported isolates [41], identical to the CPS of the related bacterium *Burkholderia mallei* [42]. The synthesis of *B. pseudomallei* and *B. mallei* CPS is challenging due to the presence of β-mannoside linkages and of the CH_2_-extension at C-6 (Figure 6). In 2016, Scott et al. [37] reported the first synthesis of hexasaccharide **52a** starting from key disaccharide fragment **53**, which was in turn synthesized from disaccharide **54**, which was assembled from building blocks **55** and **56**, convergently prepared in large scale from common intermediate **57**. This compound was obtained in seven steps from glycal intermediate **58**, which was synthesized from mannose **59** (Figure 6). The β-mannoside linkages were introduced using an indirect method, based on stereoselective β-glycosylation (ensured by 2-*O*-acyl participation on the donor) followed by C-2 epimerization. The latter step, leading to the *manno*-configuration, was performed at the disaccharide level and after each iterative coupling, through a two-step oxidation-reduction with high stereoselectivity. Hexasaccharide **52a** was covalently linked to TT and glycoconjugate **52b**, upon mice immunization, raised low but detectable levels of IgG/IgM, as determined by ELISA test. Glycoconjugate **52b**, however, was shown to stimulate production of antibodies specific for native CPS, with high functional activity correlated with protective efficacy, as observed by protection in mice following a lethal dose administration of *B. pseudomallei* [37].

## 5. *Brucella*

**Figure 7:**
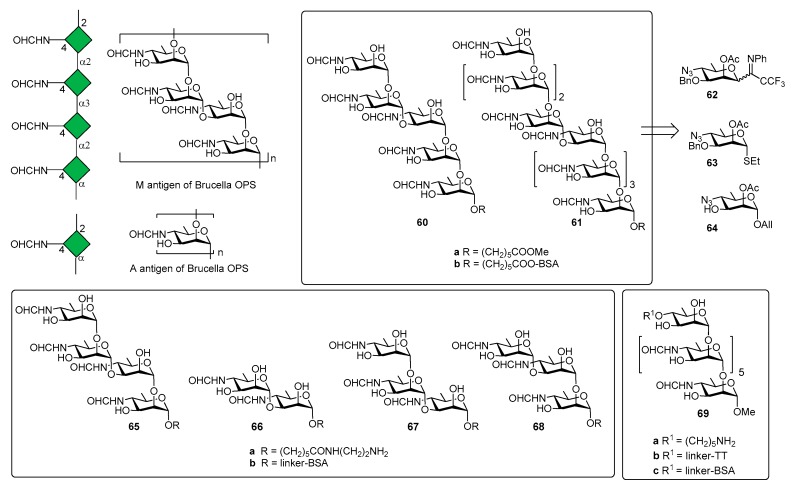
A and M antigenic determinants in *Brucella* OPS and oligomers synthesized by Bundle et al. [49].

*Brucella* is one of the world’s major zoonotic pathogens, causing brucellosis, primarily a disease of animals, such as swine, dogs, cattle, sheep, and goats [43]. Humans are infected by close animal contact or consumption of animal products (raw milk, raw milk products, or raw meat) infected by bacteria of the genus [44,45]. The genus *Brucella* comprises Gram-negative, facultative and intracellular pathogens and the current classification of recognized species is based on phenotypic characteristics, antigenic variation and prevalence of infection in different animal hosts [46,47]. The disease is not spread by human-human contact and the vaccination of animals appears as the only means for disease eradication by vaccination strategies [48]. The *O*-antigen polysaccharide domain (OPS) of *Brucella* LPS is a copolymer of two distinct homopolysaccharide sequences containing the rare sugar 4,6-dideoxy-4-formamido-α-d-mannose (-α-d-Rha*p*4NFo) [6] and simultaneously expresses two antigens, the A and M antigens (Figure 7).

Three *Brucella* antigenic phenotypes A^+^M^−^ (A-dominant), A^−^M^+^ (M-dominant) and A^+^M^+^ have been identified in *Brucella* strains [50] and antibodies (IgM) against A and M antigens have been used to detect brucellosis [51,52]. The chemical structure of A and M antigens (Figure 7) was definitively elucidated only recently [53]: a longer inner sequence of α(1,2)-linked residues constitutes the A antigen. A shorter sequence, the M antigen, consists of tetrasaccharide repeating units linked as [α(1,2);α(1,3);α(1,2)] and attached to additional copies of this tetrasaccharide or to the A antigen by an α(1,2) linkage [49].

In 2013, the Bundle group [54] reported the synthesis of pentasaccharide **60a** (Figure 7) and nonasaccharide **61a**, starting from monosaccharides **62**, **63** and **64**. The synthetic compounds were tested for antigenicity, after conjugation with bovine serum albumin (BSA) [54]. Glycoconjugate **60b** was designed to selectively exhibit the M epitope with limited cross reactivity with A-specific antibodies. The nonasaccharide conjugate (compound **61b**), containing A and M epitopes, was designed as a possible universal antigen to detect antibodies in animals or humans infected by *B. abortus*, *B. melitensis*, and *B. suis*. An ELISA test was performed with two monoclonal antibodies (YsT9-1 and Bm10) specific for the *Brucella* A and M antigens, respectively. Interestingly, nonasaccharide antigen **61a** bound A- and M-specific antibodies with equivalent avidity, whereas pentasaccharide **60a** displays a preference for the M-specific antibody, as expected. However, pentasaccharide **60a**, still displaying α(1,2)-linked residues, retained modest to good binding to A-specific mAbs. This initial result paved the way to produce a glycoconjugate vaccine that would not raise antibodies giving false positive results in diagnostic tests for infection. Indeed, the detection of specific anti-M antibodies would indicate infection by *Brucella* and not by one of the other closely related bacteria that have PS containing 1,2-linked Rha4NFo or Rha4NAc and are known to induce antibodies reactive in the serological test for brucellosis [55]. In a following work, tetrasaccharide **65a**, disaccharide **66a** and trisaccharides **67a** and **68a** (Figure 7) were synthesized to assess the largest and smallest M epitopes [56]. International standard *B. abortus* serum prepared from cattle experimentally infected with an A-dominant strain bound strongly to disaccharide-BSA conjugate **66b** and M tetrasaccharide-BSA conjugate **65b** [56]. In addition, **65b** and **66b** also showed strong binding to M-specific mAbs and weak binding with A-specific mAbs. It was also observed that antibodies raised against exclusively α(1,2)-linked Rha*p*4NFo did not bind well to the 1,3-linked disaccharide [57]. Further improvement of serodiagnosis of brucellosis came when a tether was introduced at the O-4 of the upstream residue (heptasaccharide **69a**, Figure 7) [58]. In particular, conjugate **69b** (TT) was used for mice immunization and conjugate **68c** (with bovine serum albumin, BSA) to monitor antibody responses by ELISA. Mice immunization with glycoconjugate **69b** showed that antibodies to the *Brucella* A antigen could be produced and that these antibodies didn’t react in diagnostic tests based on the M antigen. These findings were confirmed by the results of immunization studies with the OPS of *B. abortus* strain S99, which contains 98% α-(1,2) and only 2% α(1,3) linkages conjugated to tetanus toxoid. The OPS was subjected to an oxidation reaction using a procedure that concomitantly oxidized all terminal d-Rha*p*4NFo residue, essentially destroying the M epitope [58]. Immunization studies in mice showed that antibodies against the A epitope dominated. Taken together, all these studies contributed to identify the main elements for a glycoconjugate vaccine candidate for brucellosis and demonstrated that diagnostics based upon the M or A (terminal) epitopes can discriminate infected from vaccinated animals.

## 6. *Haemophilus influenzae* Type b (Hib)

**Figure 8:**
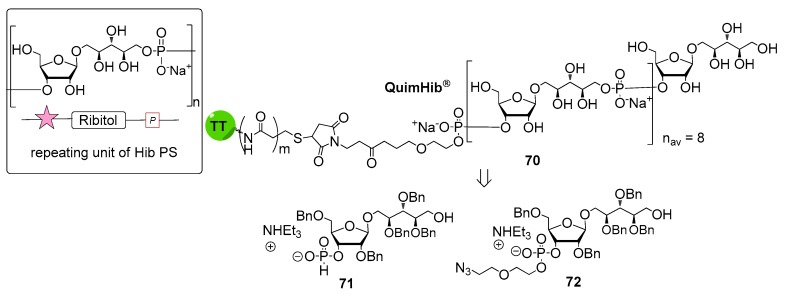
Synthesis of QuimiHib.

*Haemophilus influenzae* is a Gram-negative bacterium predominantly colonizing the human respiratory tract. *H. influenzae* strains are divided into two subgroups: unencapsulated strains, also named non-typeable (non-reactive with typing antisera) and encapsulated strains (reactive with typing antisera) comprising six serotypes: a, b, c, d, e and f. In particular, serotype b strains (*H. influenzae* b, Hib) cause severe diseases including meningitis, pneumonia and septicemia, especially in infants and children [59]. Hib CPS consists of a polymer of β-d-ribose-d-ribitol-5-phosphate (PRP) disaccharide, characterized by the presence of a phosphodiester linkage between repeating units (Figure 8).

**Figure 9:**
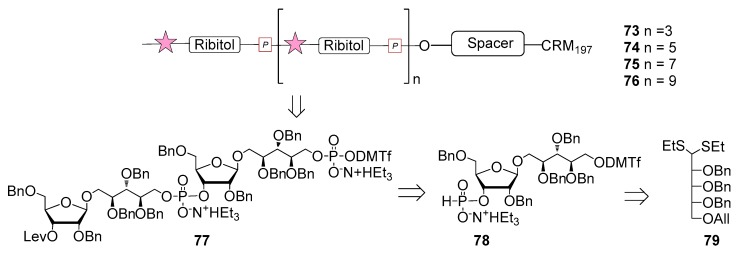
*Haemophilus influenzae* type b PRP oligosaccharides reported in [63].

The first generation of Hib vaccines, made with purified polyribosyl-ribitol phosphate, induced relatively low titers of serum antibodies, insufficient to protect children from invasive disease [60], and were replaced by Hib PS-conjugate vaccines (PedVaxHIB^®^, ActHib^®^, HibTiter^®^) [61]. Vérez Bencomo et al. developed the first synthetic glycoconjugate vaccine in 2004 [9], QuimiHib^®^ (compound **70**, Figure 8) using a one-pot polycondensation strategy starting from synthetic β-d-ribose-(1,1)-d-ribitol-5-H-phosphonate derivative **71** and the phosphodiester-linked compound **72** [9]. Final conjugation to TT gave the fully synthetic glycoconjugate vaccine QuimiHib^®^ [62], which contains a mixture of oligosaccharides with six to eight repeating units on average. Recently, Baek et al. [62] have reported the synthesis of CRM_197_ glycoconjugates of PRP oligosaccharides up to decamers (compounds **73**–**76**, Figure 9).

Oligosaccharide synthesis was performed via H-phosphonate chemistry starting from tetrasaccharide building block **77** and using a [4+4] iterative elongation strategy. Tetrameric, hexameric, octameric and decameric PRP fragments were obtained using this iterative approach, followed by the introduction of phosphodiester-linked spacer. Tetrasaccharide **77** was synthesized from disaccharide **78** (Figure 9), in turn obtained from the dithioacetal building block **79**. After conjugation to CRM_197_, immunogenicity studies with the synthetic glycoconjugates **73**–**76** were performed in a rabbit model. After immunization, sera IgG levels towards the PRP oligosaccharides were determined by glycan array analysis. Tetramer conjugate **73** and octamer conjugate **75** exhibited the highest immunogenicity, most likely indicating that four repeating units are sufficient for immunogenicity, while the hexamer conjugate **74** exhibited lower immunogenicity. This result was ascribed to the folding of the structures and to their different interaction with the immune system receptors. The authors concluded that glycoconjugates of synthetic Hib PRP are immunogenic in a rabbit model and, in particular, tetrameric conjugate **73** is a promising candidate for the design of a new glycoconjugate Hib vaccine.

## 7. *Streptococcus pneumoniae*

*S. pneumoniae*, a Gram-positive organism, is a major cause of pneumonia, otitis media, meningitis and septicemia. Various virulence determinants of pneumococci have been identified including the highly variable capsular polysaccharide (CPS), pneumolysin toxin and surface lectins. Bentley and colleagues have determined the DNA sequence of the capsular biosynthesis genes for all 90 serotypes (ST) of *S. pneumoniae* and found that each serotype has a different CPS composition [2,64]. The first generation carbohydrate-based vaccine PPV23 (Pneumovax^®^, Merck) containing the 23 most prevalent serotypes is available in the United States and in Europe, although conflicting data about its efficacy have been reported [65]. To improve the immunogenicity, glycoconjugate vaccines like PCV7 (Prevnar^®^, containing PS from serotypes 4, 6B, 9V, 14, 18C, 19F, and 23F), PCV13 (Prevnar 13™, containing PS from serotypes 4, 6A, 6B, 7F, 9V, 14, 18C, 19A, 19F, 23F, 1, 3 and 5) and PCV10 (GlaxoSmithKline’s Synflorix™, containing PS from serotypes 1, 4, 5, 6B, 7F, 9V, 14, 18C, 19F, and 23F) have been licensed and commercialized. Although *S. pneumoniae* CPS-based glycoconjugate vaccines are in current routine immunization programs and notwithstanding the increased coverage of strains, diseases caused by serotypes not included in the above vaccines can increase in the long run [64,66]. Recent efforts have been dedicated to the synthesis of antigens from *S. pneumoniae* serotypes not included in licensed formulations. Of note, glycoconjugates from synthetic fragments of *S. pneumoniae* serotype 8 have been tested in coformulation with PCV13, as reported in Section 7.6. Recently, the synthesis of the hexasaccharide repeating unit of *S. pneumoniae* serotype 12F, also not included in marketed formulations, has been reported by Seeberger et al. [67]. Meanwhile, alternative and combined approaches are emerging for vaccine development, based, for instance, on immunization with a combination of bacterial lectins and surface polysaccharides. In a recent study, the surface polysaccharide serotype 6B (PS6B) of *S. pneumoniae* was conjugated to a recombinant pneumococcal surface protein A (lectin rPspA), a highly immunogenic surface protein produced by all strains of *S. pneumoniae*, showing the ability of the novel conjugate to induce production of functional anti-rPspA1 and anti-PS6B antibodies [68].

### 7.1. S. pneumoniae Serotype 1

**Figure 10:**
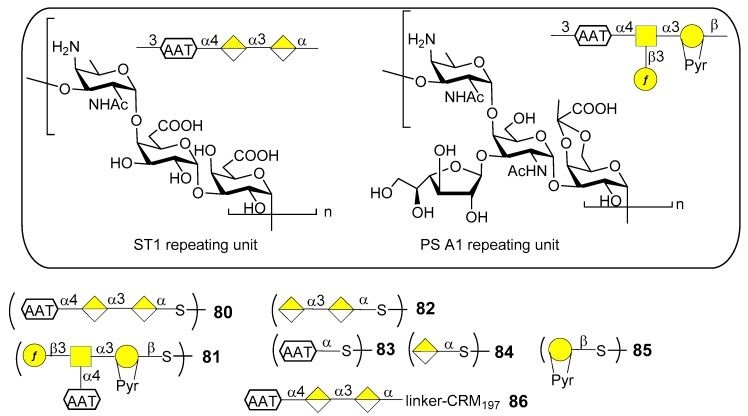
Repeating units of *Streptococcus pneumoniae* 1 CPS and *Bacteroides fragilils* PS A1 CPS and synthetic glycan fragments reported in [72].

*S. pneumoniae* serotype 1 (ST1) CPS (Figure 10) contains the rare monosaccharide 2-acetamido-4-amino-2,4,6-trideoxy-d-galactose (d-AAT) bearing a free amine at C-4. Synthetic fragments of ST1 CPS have been reported by Wu et al. [69], Christina et al. [70] and Schumann et al. [71]. In particular, Schumann et al. also contributed to the identification of the protective epitope of ST1 CPS. ST1 is one of the serotypes difficult to target by vaccination due to the low levels of functional antibodies induced by licensed glycoconjugate vaccines. This was recently [72] ascribed to the concealment of the protective epitope during chemical activation and conjugation to carrier protein. Indeed, conjugation strategies by means of reductive amination (PCV13) or 1-cyano-4-dimethylaminopyridine activation chemistry (PCV10) could lead to partial destruction of the d-AAT moieties by reaction with the free amines on this rare monosaccharide. To confirm this assumption Schumann et al. [72] synthesized and tested fragments of ST1 CPS and of the closely related *Bacteroides fragilils* PS A1 CPS (Figure 10). Synthetic oligosaccharides **80**–**85** were then subjected to glycan microarray analysis of ST1- and PS A1-directed antisera. Trisaccharide **80** bound to antibodies contained in ST1 typing serum, while disaccharide **82**, missing the d-AAT moiety, was bound in a much lower extent, revealing the importance of d-AAT for immune recognition. Neither the PS A1 repeating unit **81** nor d-AAT alone **83** or galacturonic acid alone **84** were bound. Trisaccharide **80** was then conjugated to CRM_197_ (glycoconjugate **86**) by reaction with the thiol group, thus preserving the amino group of d-AAT.

Immunization studies in rabbit models showed that glycoconjugate **86** elicited a higher immune response against trisaccharide **80**, d-AAT **83** as well as ST1 CPS compared to PCV13 or CRM_197_ alone. The antibacterial properties of sera against glycoconjugate **86** were evaluated in vitro and in vivo. In particular, flow cytometry revealed that antibodies in sera from glycoconjugate **86**-immunized rabbits bound better to ST1 bacteria than sera from PCV13-immunized rabbits. Bacterial binding correlated with serum opsonophagocytic killing capacities. Mice were passively immunized with serum of rabbits immunized with glycoconjugate **86** and then transnasally infected with ST1 pneumococci, showing fewer bacterial colonies than mice pretreated with sera from PCV13 or CRM_197_ alone-immunized rabbits. Given the importance of these findings, glycoconjugate **86** is now advancing in preclinical development for inclusion in semisynthetic vaccines covering multiple pneumococcal serotypes [72]. Interestingly, this work further demonstrates that the use of synthetic oligosaccharide antigens may be crucial to unveil hidden protective epitopes by means of site-selective protein conjugation.

### 7.2. S. pneumoniae Serotype 2

**Figure 11:**
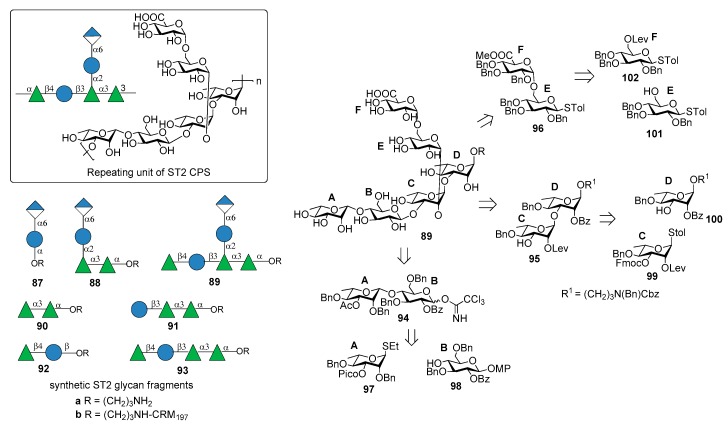
Repeating unit of *Streptococcus pneumoniae* 2 CPS and synthetic ST2 glycan fragments reported in [76].

ST2 is one of “nonvaccine serotype”, i.e., not covered by licensed PCVs based on capsular polysaccharides. It is one of the main cause of invasive pneumococcal diseases (IPD) responsible for pneumonia, septicemia, meningitis, and otitis media in many countries in Asia [73] and Central America [74]. The structure of ST2 CPS is composed of a hexasaccharide repeating unit illustrated in Figure 11 [75].

Emmadi et al. [76] reported the synthesis of the repeating unit of ST2 CPS and of series of synthetic glycans containing portions of the ST2 CPS (compounds **87a**–**93a**), in order to identify the protective oligosaccharide epitope. Hexasaccharide **89a** (one repeating unit) was synthesized from disaccharides **94**, **95**, **96** via a [2+2+2] glycosylation strategy (Figure 11). These disaccharide units were in turn synthesized from l-rhamnose and d-glucose building blocks **97**–**102** (Figure 11). The β-rhamnosidic linkage in **94** was incorporated by installing a remote C3 picoloyl group on rhamnosyl thioglycoside **97** for hydrogen-bond-mediated aglycon delivery. The 1,2-*cis* linkage between glucose building blocks **101** and **102** was formed by in situ anomerization, by converting **102** to the corresponding glycosyl bromide and then by reatcion with **101** in the presence of TBAI. Glycan microarrays containing oligosaccharide fragments **87a**–**93a** were used to screen human and rabbit sera specific to serotype 2 CPS and to identify epitope hits. These experiments demonstrated that the α-d-GlcA-(1→6)-α-d-Glc-(1→2) branch is important to have strong specific antibody binding. Hexasaccharide **89a** was conjugated to CRM_197_ and used for mice immunization producing very high titers of CPS-specific opsonizing antibodies that efficiently fix complement and promote killing of pneumococci by phagocytic activity. An in vivo experiment to evaluate the vaccine involved subcutaneous immunization of mice that were infected with highly virulent ST2 strain NCTC7466. Neoglycoconjugate hexasaccharide-CRM_197_ **89b** stimulated a T cell-dependent B cell response that induced CPS-specific antibodies resulting in the reduction of the bacterial infection in lung tissues and blood.

### 7.3. S. pneumoniae Serotype 3

**Figure 12:**
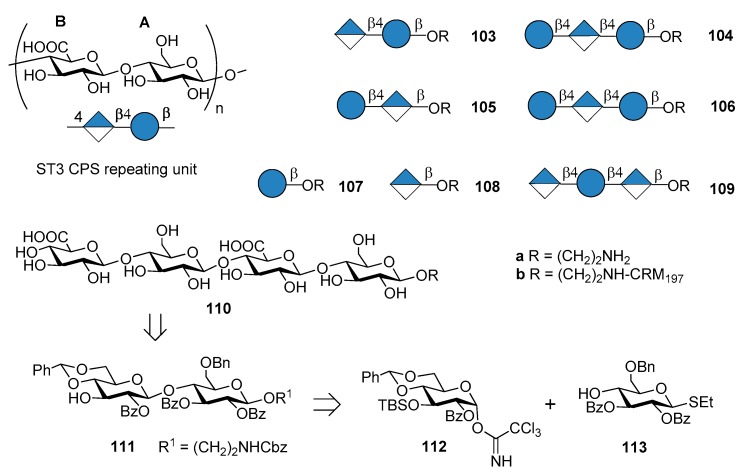
Repeating unit of *Streptococcus pneumoniae 3* CPS and synthetic ST3 glycan fragments reported in [81].

The commercial anti-pneumococcal glycoconjugate vaccine PCV13 includes *S. pneumoniae* serotype 3 (ST3). However, ST3 glycoconjugate contained in PCV13 has shown an atypical immunogenicity, ascribed to the abundant CPS expression on the capsule and to a weakened booster response leading to hyporesponsiveness (inability of the individual to mount an immune response after booster vaccination) [77,78]. Indeed, the levels of pre-existing ST3-specific antibody were found to be negatively correlated with the B cell memory response to a booster dose of PCV13 containing ST3 glycoconjugate [79]. This behavior has been associated with a lack of protection against acute otitis media [80]. As a consequence, one can assume that pure synthetic antigenic structures, designed on the basis of antibody binding specificities, could improve immunogenic properties of ST3 CPS conjugates. Synthetic oligosaccharides based on ST3 CPS repeating units have been already reported by Benaissa-Trouw et al. in 2001 [80] and they have been proven to protect mice against lethal intraperitoneal challenge with ST3 pneumococci. Recently, Parameswarappa et al. [81] reported the synthesis of a library of oligosaccharides, compounds **103a**–**110a** and their corresponding CRM_197_ conjugates **103b**–**110b** (Figure 12).

The synthesis of the fragments was achieved from disaccharide **111**, in turn obtained from two differentially protected glucose building blocks **112** and **113**. Tetrasaccharide **110a** was synthesized with a [2+2] strategy in 13% overall yield from **112** and **113**. The synthetic ST3 oligosaccharides potentially contained the minimal protective glycan epitope (Figure 12). Glycan arrays containing the different fragments were used to screen human sera for antibodies and to define the recognition site of two protective ST3-specific monoclonal antibodies (mAbs). Tetrasaccharide **110a** contains the protective epitope of both mAbs and was selected for further immunogenicity studies. The CRM_197_ conjugate **110b** elicited protective immunity as evidenced by opsonophagocytosis assays and mice immunization experiments against experimental pneumonia caused by transnasal infection with ST3 strain PN36. Formulation of the protective epitope has to be further evaluated to elicit optimal long-term immunity.

**Figure 13:**
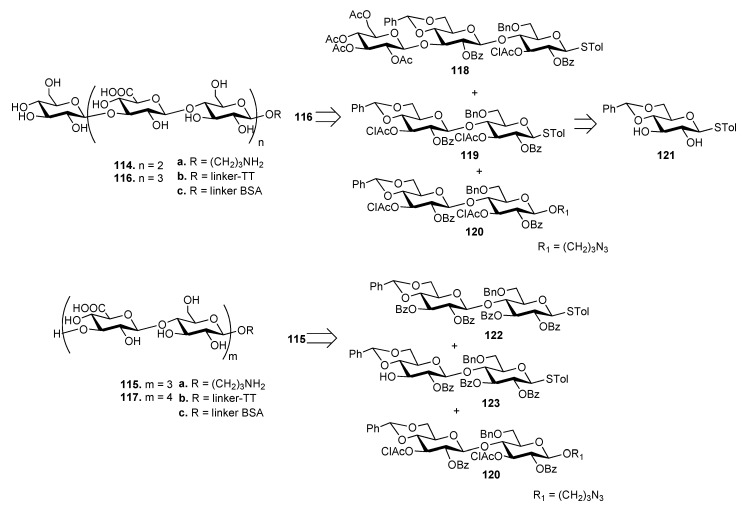
Synthesis of ST3 oligosaccharides and glycoconjugates from [82].

The synthesis of ST3 CPS oligosaccharides **114**–**117** (Figure 13) has been recently reported by Xiong et al. [82] These oligosaccharides were also designed to have different sugar residues, Glc (**114** and **116**) or GlcA (**115** and **117**) at the upstream end. As an example, heptasaccharide **116** was synthesized by a 3+[2+2] glycosylation strategy from trisaccharide **118** and disaccharides **119** and **120**, all achieved from common precursor **121**. Hexasaccharide **115** was synthesized by a [2+2]+2 strategy from disaccharides **122**, **123** and **120**. The oligosaccharides were designed to expose a free amino group at their downstream ends (**114a**–**117a**) to allow conjugation with tetanus toxoid (TT) (**114b**–**117b**) and BSA (**114c**–**117c**) carrier proteins. [83]. TT conjugates **114b**–**117b** and free oligosaccharides **114a**–**117a** were injected in mice and the obtained antisera were analyzed by ELISA using BSA conjugates **114c**–**117c** as capture antigens. Antisera derived from mice immunized with TT conjugates **114b**–**117b** contained significantly higher specific antibodies compared to **114a**–**117a**. In particular, antibody titers induced by **114b** and **115b** were significantly higher than those induced by **116b** and **117b**, showing that the chain length of ST3 CPS oligosaccharides influences the immunological properties and that longer oligosaccharides are not necessarily better haptens.

### 7.4. S. pneumoniae Serotype 4

**Figure 14:**
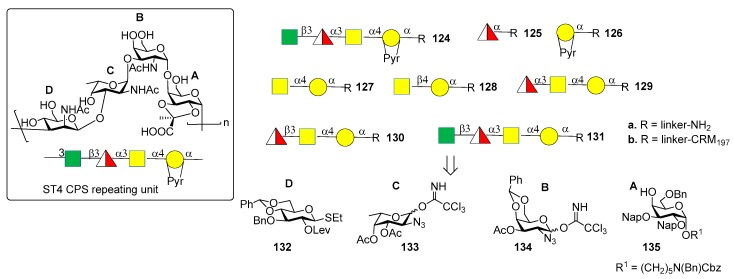
Repeating unit of ST4 CPS and synthesis of ST4 CPS fragments in the pyruvalated form and the nonpyruvalated variant, as reported in [84].

The CPS of *S. pneumoniae* serotype 4 (ST4) contains a rare and labile substituent, the trans-2,3-(S) cyclic pyruvate ketal modified galactose (residue A, Figure 12). The ST4 repeating unit is a tetrasaccharide made of [3)-β-d-Man*p*NAc-(1→3)-α-l-Fuc*p*NAc-(1→3)-α-d-Gal*p*NAc-(1→4)-α-d-Gal*p*-2, 3-(S)-Pyr-(1→] (Figure 14).

Recently, the Seeberger group [84,85] reported the synthesis and immunological evaluation of fragment **124a**, corresponding to the repeating unit, and shorter oligomers **125a**–**131a** with and without the pyruvate ketal, demonstrating the importance of the trans-2,3(S)-pyruvate ketal in the ST4 epitope. In particular, the synthetic fragments were obtained with a linear glycosylation approach from building blocks **132**–**135** (Figure 13). Of note, for the installation of 1,2-cis linkages, glycosylation of galactose **135** with donor **134** occurred with good stereoselectivity of the newly formed glycosidic linkage (α:β = 7:1). On the other hand, installation of the final β-*manno* linkage in unit D was accomplished using an indirect two-steps method. Indeed, exclusive β-glucosylation was achieved with donor **132** using NIS and TfOH as promoters. The following 2-OH activation and amination established the desired *manno* configuration at C-2. Glycan arrays showed that ST4-directed antibodies in the human reference serum (serum 007sp) [85] recognized both pyruvate-dependent and pyruvate-independent epitopes. Oligosaccharide **124a** showed the highest antibody affinity and cross-reactivity to ST4 CPS in mice and humans immunized with the natural CPS. Human serum 007sp contains antibodies recognizing also non-pyruvalated oligosaccharides **129a** and **131a**. Thus, it was hypothesized that non-pyruvalated epitopes could be present in the natural CPS, although non-pyruvalated epitopes could be less immunogenic than pyruvalated epitopes, as indicated by lower antibody binding signals to **129a** and **131a** compared to **124a**. To verify this behavior, two selected CRM_197_ conjugates of non-pyruvalated ST4 oligosaccharides, **129b** and **131b**, were used for mice immunization. The raised antibodies did not recognize the natural polysaccharide on the surface of ST4 bacteria. This result confirmed that the pyruvate motif on the oligosaccharide is needed for cross-reactivity with the native CPS.

### 7.5. S. pneumoniae Serotype 5

**Figure 15:**
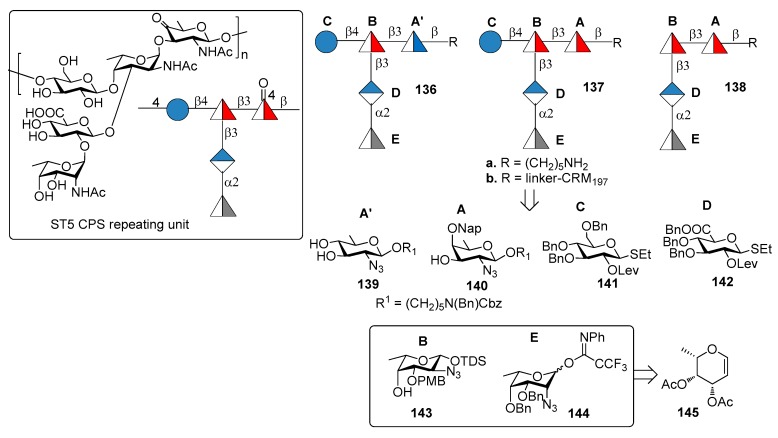
Repeating unit of ST5 CPS and synthetic glycan fragments reported in [89].

Serotype 5 (ST5) is the fifth most prevalent serotype of *S. pneumoniae* and is included in the PCV10 and PCV13 [86]. ST5 CPS repeating unit (Figure 15) [87] contains a branched *N*-acetyl-l-fucosamine (l-Fuc*p*NAc) linked to d-glucose (d-Glc) and d-glucuronic acid (d-GlcA) and two rare deoxyamino sugars: the ketoamino sugar 2-acetamido-2,6-dideoxy-d-xylose-hexos-4-ulose (Sug*p*) and the *N*-acetyl-l-pneumosamine (l-Pneu*p*NAc), which is α(1→2) linked to d-Glc*p*A. During CPS isolation and purification for the production of the glycoconjugate vaccine, the keto group of Sug*p* can be partially or fully reduced to form a mixture of ST5 CPS components with decreased immunogenicity compared with the native ST5 CPS [88].

Recently, Lisboa et al. [89] reported the synthesis of ST5 CPS fragments **136a**–**138a** starting from six differentially protected monosaccharide building blocks **139**–**144** (Figure 15). In particular, l-fucosamine acceptor **143** and l-pneumosamine donor **144** were both synthesized from l-fucal **145** via an azido-phenylselenation reaction on the double bond. Among the oligomers synthesized, oligomer **136a** contains *N*-acetyl-d-quinovosamine (A’, d-QuiNAc) in place of of Sug*p* (A), displaying a hydroxyl group at C-4 in place of the labile carbonyl occurring in the native ST5 CPS. Seeberger group [89] uncovered the protective ST5 CPS epitope using a combination of glycan microarray-based mAb generation and immunological evaluation performed in rabbit models. These experiments showed that the rare aminosugar l-PneuNAc, as well as the branching, are essential for antibody recognition and avidity. Interestingly, it was also demonstrated that CRM_197_ glycoconjugate **136b**, containing d-QuiNAc, induced higher antibody titers and opsonic activity compared to native ST5-CRM_197_ conjugate contained in PCV13 vaccine. Special care should be taken, however, in the interpretation of the results obtained with such different vaccination modalities, i.e., a 13-valent vaccine vs. a monovalent synthetic vaccine. The latter indeed contains only one type of carbohydrate which is administered in much higher amount in comparison to the same carbohydrate contained in PCV13. Nevertheless, this result suggests the possibility for the replacement of labile functional groups, generating manufacture issues, with stable functional groups that do not affect the immunogenic properties of glycoconjugates.

### 7.6. S. pneumoniae Serotype 8

**Figure 16:**
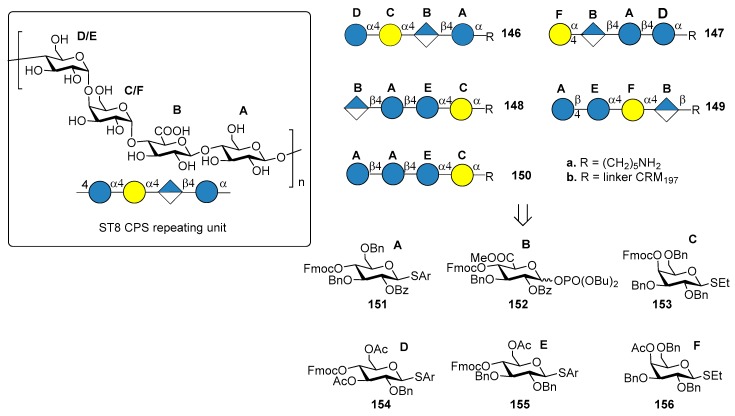
Repeating unit of *Streptococcus pneumoniae* 8 *CPS* and synthetic ST8 glycan fragments reported in [92].

ST8 is part of the first-generation polysaccharide vaccine PPV23, but it is not included in glycoconjugate vaccines PCV7, PCV13 and PCV10. Many clinical ST8 isolates were found resistant to antibiotics like erythromycin, clindamycin, tetracycline and ciprofloxacin [90]. Furthermore, this multiresistant serotype is a major cause for concern in HIV-infected patients, where its occurrence is significantly more frequent [91]. ST8 CPS consists of a linear tetrasaccharide repeating unit (Figure 16) sharing a common cellobiuronic acid disaccharide [β-d-GlcA-(1→4)-β-d-Glc] with ST3 CPS (BA sequence, Figure 12).

Schumann et al. [92] reported the preparation of tetrasaccharide fragments of ST8 CPS **146a**–**150a**, to identify the minimal protective glycan epitope. The four tetrasaccharides were synthesized by automated glycan assembly, using solid-phase oligosaccharide synthesis, starting from building blocks **151**–**156**. Glycan microarray containing all ST8 CPS frameshifts led to the identification of one tetrasaccharide frameshift (BAEC, **148a**) that was preferentially recognized by a protective mAb, a murine immunoglobulin M (IgM) against native ST8 CPS [93]. Conjugation with CRM_197_ of the tetrasaccharide **148a** gave the ST8 glycoconjugate **148b**, which was used for immunization of mice and rabbit models. Interestingly, although cellobiuronic acid disaccharide conjugate **103b** (BA, Figure 12) conferred protective immunity against ST3 [81], no ST3-directed immune response was found after mice immunization with conjugate **148b** (BAEC), probably because of the different presentation of cellobiuronic acid in the ST8 sequence. Conjugation with CRM_197_ and coformulation with PCV13 of either tetrasaccharide **148a** or its congener tetrasaccharide **150a** (AAEC), containing a d-Glc residue in place of the naturally occurring d-GlcA, led to a new vaccine which conferred protective immunity in rabbits against all the 14 *S. pneumoniae* serotypes. This achievement confirms the possibility of adding synthetic oligosaccharide antigens to existing vaccines with the aim of expanding current formulations and replacing serotypes that are not efficiently targeted.

### 7.7. S. pneumoniae Serotypes 14 and 19F

**Figure 17:**
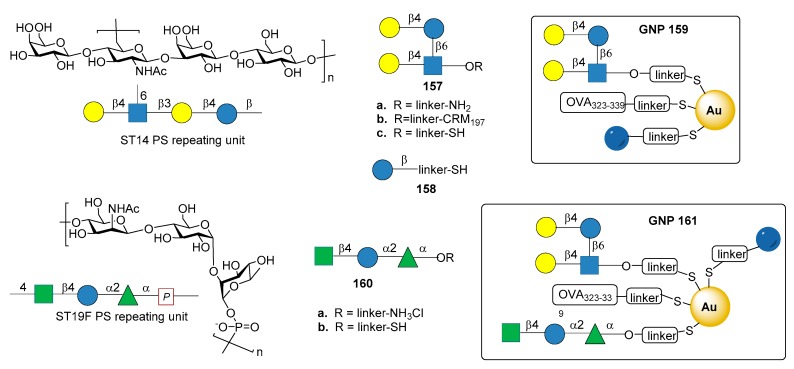
Repeating unit of *S. pneumoniae* type 14 (ST14) and *S. pneumoniae* type 19F (ST19F), and their synthetic fragment conjugated to gold nanoparticles reported in Reference [96] and in Reference [98].

Among the synthetic glycoconjugate vaccines for *S. pneumoniae*, gold nanoclusters have been recently explored, highlighting the potential of these carriers for the development of synthetic vaccines [94,95]. In particular, glyconanoparticles bearing the synthetic tetrasaccharide epitope of *S. pneumoniae* type 14 (ST14) PS have been recently reported [96]. ST14 PS consists of repeating units of the tetrasaccharide (6)-[β-d-Gal*p*-(1→4)-]β-d-Glc*p*NAc-(1→3)β-d-Gal*p*-(1→4)β-d-Glc*p*-(1→)_n_ (Figure 17) [97].

The synthetic branched tetrasaccharide Gal-Glc-(Gal-)GlcNAc (**157a**), synthesized and studied by Mawas et al. [99] was identified as the smallest structure producing protective antibodies against ST14 when conjugated to CRM_197_ protein (glycoconjugate **157b**) [100]. Tetrasaccharide **157c**, derivatized with a terminal thiol for nanoparticle functionalization, was conjugated together with the T cell-stimulating ovalbumin peptide (OVA 323–329) and d-glucose fragment **158** to produce small (2 nm) hybrid gold glyconanoparticles **159** (GNPs, Figure 17). Immunogenicity studies in mice showed that **159** induced the production of specific IgG antibodies against ST14 PS. The presence of OVA 323–339 peptide was necessary for the induction of high affinity IgG antibodies, while the T cell epitope did not raise anti-OVA 323–339 peptide antibodies, thus avoiding the risk of epitope suppression. Sera obtained from mice immunized with **159** with a ratio of tetrasaccharide:Glc:OVA 323–339 = 45:50:5 were able to opsonize ST14 bacteria, although less efficiently than sera from mice immunized with native ST14 PS conjugated to CRM_197_. These results make **159** a promising *S. pneumoniae* type 14 vaccine candidate.

In another recent study [98], gold glyco-nanoparticles (GNP) were prepared with synthetic oligosaccharide fragments corresponding to the repeating units of *S. pneumoniae* CPS type 19F and 14. In particular trisaccharide **140a**, corresponding to ST19F repeating unit [β-d-Man*p*NAc-(1→4)-α-d-Glc*p*-(1→2)-α-l-Rha*p*-(1→] (Figure 17), was prepared according to procedures described in the literature [101,102] and derivatized as thiol-ending ligand **160b**. Tetrasaccharide **157c** (fragment of ST14), trisaccharide **160b** (fragment of Pn19F), d-glucose fragment **158** and OVA 323–339 peptide were loaded onto GNPs (**161**, Figure 17) in different ratios. After mice immunization, GNPs **161** enhanced the production of specific IgG antibodies toward ST14 PS, while no IgG antibodies against ST19F PS were elicited. In particular, the titers of specific IgG antibodies towards ST14 polysaccharide raised by **161** were higher than the titers elicited by GNPs exclusively displaying ST14 (**159**), and comparable with commercially available PCV13. Of note, this work explored the effect on the immunological response of glyconanoparticles displaying two carbohydrate epitopes from different bacterial serotypes.

## 8. *Group A Streptococcus*

**Figure 18:**
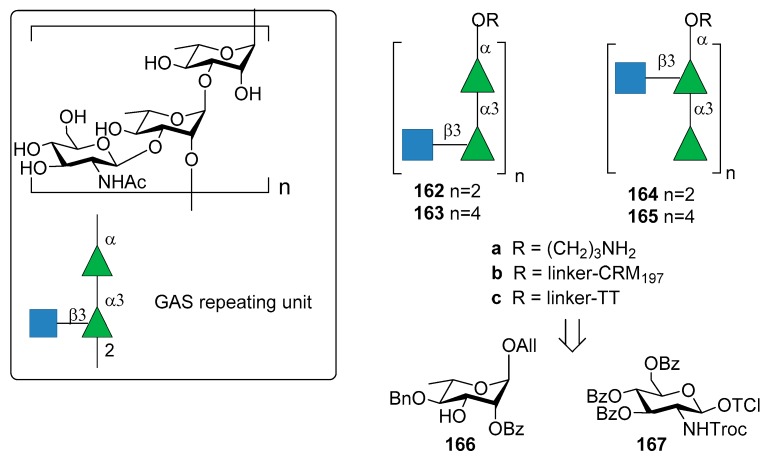
Repeating unit of the cell wall PS of GAS and synthetic fragments reported in [104].

*Group A Streptococcus* (GAS) is a Gram-positive microorganism causing post-sequelae autoimmune infections including rheumatic heart disease. The main driver of autoimmunity is the surface-anchored GAS M polymorphic proteins [103]. Indeed, formulated multivalent M protein-based vaccines were tested in animal and human models but they are protective only for the serotypes included in formulation. For this reason, the identification of a common protective antigen is highly desirable. Due to its prominence in the GAS cell wall and its conservation across all GAS strains, the Lancefield group A carbohydrate (GAC) has been considered as a potential antigen for a universal GAS vaccine [103]. The Lancefield group A carbohydrate (GAC) consists of a α-l-Rha*p*(1→3)-α-l-Rha*p*(1→2)-[β-d-Glc*p*NAc]-(1→3) repeating unit (Figure 18).

Increasing concerns regarding autoreactivity of antibodies that recognize the native GAC GlcNAc side chain (anti-GlcNAc monoclonal antibodies) [105], however, have been supported by recent studies [106,107]. Cross-reactivity (especially in heart or brain tissues) of anti-GlcNAc mAb was hypothesized almost twenty years ago [108] and it is still a crucial point of discussion, as well as the role of polyrhamnose on the immunogenicity. More recently, Henningham et al. [109] reported that the relative contribution of GlcNAc side chains to the innate immune resistance of GAS varies among strains and that GlcNAc side chain is not a universal GAS virulence factor in animal models. In 2010, Kabanova et al. [104] reported the synthesis of two sets of hexasaccharide- and dodecasaccharide-CRM_197_ conjugates **162b**–**165b** (Figure 18) and compared their immunogenicity with the native GAC-CRM_197_ conjugate. All oligomers **162a**–**165a** were synthesized starting from building blocks **166** and **167**. Of note, the GAC isolated from bacterial fermentation was found to be contaminated with polyrhamnose variant species. The synthetic oligosaccharide conjugates **162b**–**165b** showed similar immune response in mice compared to GAC conjugate against two GAS isolates of M1 and M23 serotypes. A saccharide chain length of six (the minimal size of the antigen) was found to be sufficient to elicit protective antibodies.

More recently, Auzanneau et al. [110] reported the synthesis of hexasaccharide **164a** and its conjugation to TT carrier protein to give **164c**. Previously reported conformational analysis had shown that the branch-point in the trisaccharide repeating motif was important for antibody recognition in the antibody-ligand complex [111]. Epitope mapping of a branched trisaccharide and a doubly-branched hexasaccharide by saturation transfer difference NMR methods [112] confirmed the importance of the branched trisaccharide epitope that was studied in binding experiments with a mouse monoclonal antibody. The immunogenicity of the synthetic hexasaccharide–TT conjugate **164c** was confirmed by primary (IgM) and secondary antibody (IgG) responses, with anti-hexasaccharide titers that increased after booster immunizations to mice. These titers were similar to those obtained with the native GAC–TT conjugate.

## 9. Group B Streptococcus

**Figure 19:**
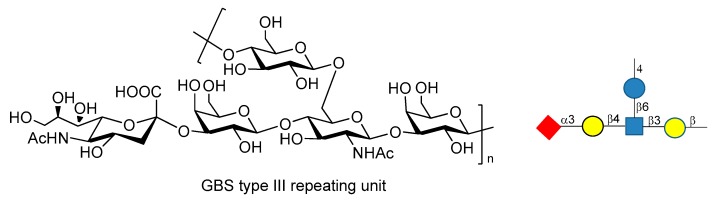
GBS PSIII repeating unit.

*Streptococcus agalactiae* or Group B Streptococcus (GBS) is the leading cause of invasive infections in pregnant women [113], newborns, and elderly people, resulting in pneumonia, sepsis and meningitis [114,115]. GBS is a multiserotype Gram-positive bacterium that expresses Lancefield group B polysaccharide as a major virulence factor. Ten different serotypes of GBS PS have been characterized (Ia, Ib, II, III, IV, V, VI, VII, VIII, IX), but five of them (Ia, Ib, II, III and V) account for the vast majority of the disease [116]. Chemical synthesis of the repeating unit of some of these serotypes (types Ia, II, V) have been reported in recent years by Guo and Gao groups [117,118,119]. These works may be useful for the synthesis of other fragments of GBS PS and for further investigations, like antigenicity and immunological studies. GBS type III repeating unit is composed of →4-β-d-Glc*p*-(1→6)-β-d-Glc*p*NAc-[α-NeuNAc-(2→3)-β-d-Gal*p*-β-(1→4)]-(1→3)-β-d-Gal*p*-(1→ (Figure 19).

Baker et al. [113,120] reported that GBS PSIII conjugated to TT carrier protein resulted in high tolerance when administered to pregnant women, raising highly specific IgG Abs titers which were transferred through the placenta to infants. Chemical synthesis of fragments of PSIII and of related desialylated fragments have been reported [121,122,123,124]. Conformational studies and molecular dynamics simulations [125,126] showed high flexibility of GBS PSIII, as it adopts a partial helical conformation thanks to the presence of α-NeuNAc-(2→3)-β-d-Gal*p*-β-(1→4) branch, while without the sialic acid residues a random coil conformation is preferred. In particular, it has been shown that there is a specific interaction between the sialic acid residues and the glucosyl and galactosyl backbone which influences the orientation of the side chain and the backbone conformation [125,126]. These behaviors have been rationalized hypothesizing the existence of an extended conformational epitope. A recent study carried out by Adamo et al. [127] showed that synthetic fragments of GBS PSIII conjugated to CRM_197_ are recognized by polyclonal PSIII specific serum and that the presence of the branch is a structural relevant motif for the recognition of anti-PSIII antibodies. Even if these *neo*-glycoconjugates can’t still be considered vaccine candidates, this promising result paves the way to the use of synthetic GBS PSIII oligosaccharide as tools to study their detailed molecular interactions with anti-PSIII mAbs.

## 10. *Salmonella Typhi*

**Figure 20:**
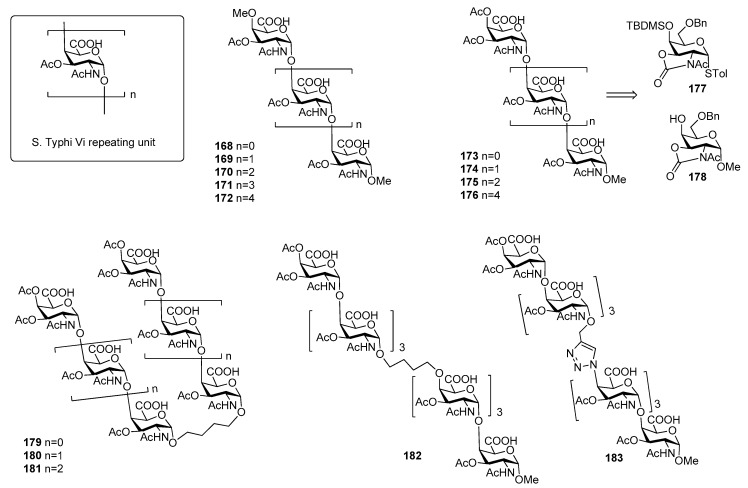
Repeating unit of *S. Typhi* Vi CPS and synthetic fragments reported in [140,141,142].

*Salmonella enterica serovar Typhi*, generally termed *Salmonella Typhi (S. Typhi)* is a highly invasive encapsulated Gram-negative bacterium, responsible for typhoid fever, a systemic infection mostly diffused in less-developed geographic areas, lacking proper sanitary conditions. Infections occur generally via consumption of contaminated food and water. Global estimates of typhoid fever burden range between 11 and 21 million cases and approximately 128,000 to 161,000 deaths annually [128], with a peak incidence in individuals from early childhood to 15 years old [129]. Clinical diagnosis of the infection is difficult due to the often non-specific symptoms of typhoid fever that can be confused with other common febrile illnesses [130] and due to serological tests that often give false-negative and false-positive results [131,132]. *S. typhi* capsule contains three antigens: the H antigen is a heat sensitive protein of the peritrichous flagellae, while the O or somatic antigen is a cell-wall lipopolysaccharide. The Vi antigen is the capsular polysaccharide which overlies the O antigen. The *Vi* antigen plays a crucial role in the modulation of early inflammatory responses during *S. Typhi* infections [133,134] and represents the basis for the formulation of vaccines against this bacterium [134,135,136]. It is called Vi (“Virulence”) antigen due to its ability to enhance *S. Typhi* virulence [137,138]. It is an anionic polymer composed by α-(1→4)-linked *N*-acetyl galactosaminuronic acid repeating units predominantly *O*-acetylated at position 3 (Figure 20). The degree of 3-*O*-acetylation ranges from 60% to more than 90% in some strains and the immunogenicity of Vi antigen is closely related to its degree of *O*-acetylation [139]. The carboxylic acids are less exposed and partially shielded by the *O*-acetyls and this can explain the minor effect upon the immunological properties observed after reduction of the carboxylic acids [139].

Although pure polysaccharide vaccines based on the purified Vi antigen have been proven effective in adults, they have been so far ineffective in infants (especially children younger than 5 years of age), in the elderly and immunocompromised individuals [143]. The coupling of *S. Typhi* CPS fragments to carrier proteins (rEPA, TT, CRM_197_) produced glycoconjugate vaccines able to elicit a T cell dependent immune response [144,145]. In particular, the conjugate vaccine Vi-TT was recently found effective in the prevention of typhoid fever in a phase 2b trial and proven to be safe and highly immunogenic [146]. The injectable Vi-TT conjugate vaccine (TCV) is currently licensed and recommended by WHO for children from 6 months of age and adults up to 45 years of age. Synthetic oligomers of Vi antigen were first reported in the literature by Sinaÿ and coworkers [140]. In particular, Vi oligosaccharides up to hexasaccharide **168**–**172** (Figure 20) bearing an unnatural *O*-methyl group both at the C4 position of the upstream residue and at C-1 position of the downstream residue have been synthesized, thus precluding protein conjugation [140]. Recently, Ye and co-workers [141] reported the synthesis of Vi oligomers **173**–**176** as methyl glycosides containing an unnatural acetyl group at C-4 of the upstream residue (Figure 20). In particular, *N*-acetyloxazolidinone-containing glycosyl donor **177** and acceptor **178** were used to direct alpha stereoselectivity during glycosylation reactions. ELISA competitive assays showed that synthetic tri- and tetra-saccharides **174** and **175** had improved antigenic activities in comparison to Sinaÿ fragments [140]. The authors speculated that improved affinities could be ascribed to the presence of an acetyl group at C-4 of the upstream residue in place of the methyl ether present in Sinaÿ’s structures. More recently, Ye et al. [142] synthesized a series of Vi pseudo-oligosaccharides **179**–**183** (Figure 20) conjugated by carbon chain spacers through olefin cross metathesis or by the 1,2,3-triazole moiety through Huisgen cycloaddition reaction. The binding affinities to anti-Vi antibodies of proposed mimics **179**–**183** were investigated, showing that the affinity of divalent compounds was generally comparable to the monovalents of the same length. For example, the affinity of **181**, containing the butylene linker did not increase significantly when compared with that of monovalent tetrasaccharide. Heterodimer **182**, which mimics the native Vi antigen with the similar chain-elongation direction did not result in improved antigenicity, perhaps suggesting that longer Vi oligomers are needed for higher affinity.

**Figure 21:**
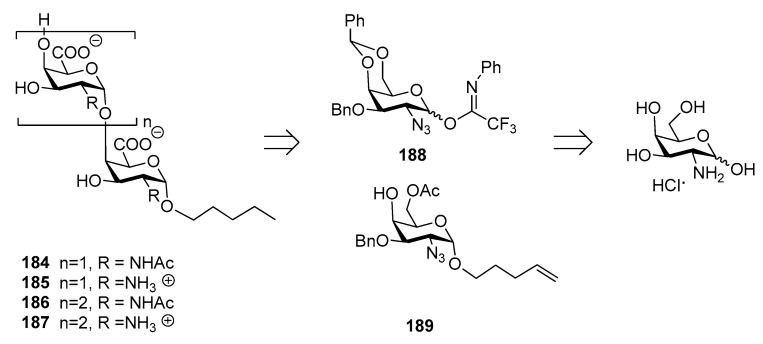
Retrosynthetic analysis of di- and trisaccharide fragments of *S. Typhi* Vi CPS, reported in Reference [147].

Recently, the synthesis of di- and trisaccharide fragments of *S. Typhi* Vi capsular polysaccharide analogues and their zwitterionic counterparts has been accomplished by the Lay group (Figure 21) [147]. These fragments were composed of *N*-acetylgalactosaminuronic acid repeating units non-acetylated at position 3 (Figure 21). The synthetic strategy was designed in order to obtain the two distinct series of oligomers **184**–**187** (2-acetamido derivatives and their zwitterionic analogues) from common building blocks, donor **188** and glycosyl acceptor **189**, in turn synthesized from commercially available d-galactosamine hydrochloride. Glycosylation reaction of acceptor **189** with donor **188** gave exclusively the desired α (1,4) disaccharide and the same 1,2-cis stereoselective outcome was observed for the trisaccharides.

ELISA tests showed that oligosaccharides **184**–**187** were recognized by specific anti-Vi polyclonal antibodies in a concentration-dependent manner with similar efficacies, lower than the natural Vi polysaccharide. This might be related to the short chain length of the synthetic fragments and to the lack of the 3-*O*-acetyl group, which has been reported as being important for the immunogenicity [139].

## 11. *Pseudomonas aeruginosa*

*Pseudomonas aeruginosa* is an opportunistic Gram-negative bacterium that can cause hospital-associated infections, often life-threatening in critically ill patients [148]. Cystic fibrosis patients often become infected with *P. aeruginosa* in chronic lung infections [149]. *P. aeruginosa* encodes several multidrug efflux pump genes [150] and has acquired multiple resistance mechanisms to most antibiotic classes, selected by years of antibiotic treatments [151]. In the past few years, new approaches such as the administration of anti-bacterial monoclonal antibodies are being investigated for the prevention or treatment of *P. aeruginosa* infections [152]. In a recent study, *P. aeruginosa* bloodstream infection isolates from patients with acute *P. aeruginosa* infections were analyzed for the ability to express PcrV, a type 3 secretion protein, and Psl exopolysaccharide, an important component of the microbial biofilm extracellular matrix [153]. The study showed that the majority of isolates expresses PcrV and Psl. However, most of the patient’s sera lacked IgG and functionally active responses to these targets. These findings suggest that Psl can shield the bacterium from the host immune response, allowing the survival of the bacterium [153]. In particular, Psl is a serotype-independent antigen anchored to the cell surface in a helical pattern, an organization that can be crucial for cell–cell interactions and to engage in interaction with other biofilm-initiating components [154,155].

**Figure 22:**
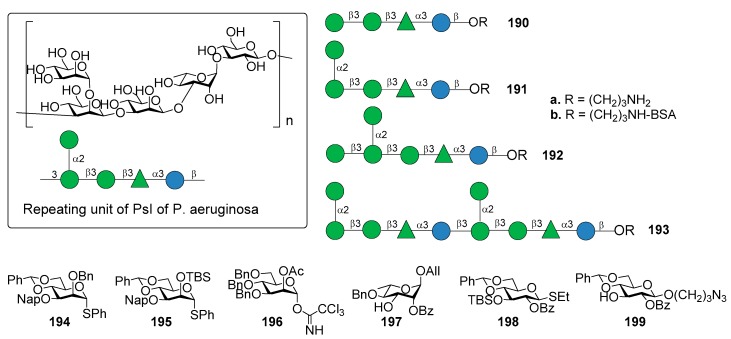
Repeating unit of PsI of *P. aeruginosa* and synthetic fragments reported in Reference [158].

Di Giandomenico et al. [156] reported the identification of mAbs, classified in class I, II, and III antibodies, binding three different epitopes of Psl, as suggested using competition antibody binding assays. The mAbs possessed opsonophagocytic killing activity and anti–cell attachment activity. In particular, class I mAb were shown to be the most functionally active and protective anti-Psl antibodies against *P. aeruginosa* [156]. The repeating unit of PsI of *P. aeruginosa* is the pentasaccharide shown in Figure 22, as determined by Kocharova et al. [157]. The chemical synthesis of different fragments of PsI, tetra-, penta-, hexa- and decasaccharide **190a**–**193a** was reported starting from building blocks **194**–**199** (Figure 22) [158].

The synthetic strategy dealt with the stereoselective glycosylation of mannosides and the formation of two 1,2-cis mannosides, one of which is also extended at C-1, C-2, and C-3 in a crowded 1,2,3-cis configuration. In particular, 4,6-*O*-benzylidene protected mannosyl donors [159] **194** and **195**, modified by a C-3 Nap ether and a C-2 silyl ether, respectively, provided optimal 1,2-cis stereoselectivity in the glycosylation reactions. On the other hand, mannosyl donor **196**, functionalized with a participating acetyl ester at C-2, resulted suitable for the preparation of 1,2-trans mannosides. Compounds **190a**–**193a** were used to identify the epitope requirements of monoclonal antibodies of class I, II, and III, showing some new insights about immune recognition of *P. aeruginosa* Psl exopolysaccharide [158]. Oligosaccharides **190a**–**193a** were conjugated to BSA (**190b**–**193b**) to facilitate coating of ELISA plates followed by testing reactivity with an antibody that bound each epitope class. The class II mAb reacted potently with all oligosaccharides, suggesting that the epitope for this class resides within tetrasaccharide **190b** and does not require the 1,2-cis mannoside of compound **191b**. The class III antibody did not bind tetra- (**190b**) or pentasaccharide BSA-conjugate (**191b**). On the contrary, it showed weak affinity to glycoconjugate **193b** and strong affinity to glycoconjugate **192b**, suggesting that the terminal glucoside contained in glycoconjugate **192b** is required for optimal binding. The class I antibody did not bind any of the oligosaccharides, suggesting the possibility that the class I mAb binds to a conformational epitope of PsI or to a substructure yet to be determined. The identification of this epitope could provide an attractive lead compound for the development of a synthetic Psl-based vaccine for *P. aeruginosa*.

## 12. *Neisseria meningitidis*

*Neisseria meningitidis* is a Gram-negative bacterium that colonizes the mucous membranes of humans. Meningococcal meningitis and sepsis are severe diseases that kill children and young adults within hours despite the availability of effective antibiotics. Mortality rates and permanent disability, like amputation, hearing loss and neurologic deficiency associated with *N. meningitidis* infections are high, even in countries where optimal health care practices are in place [160]. Among the 12 serogroups of *N. meningitidis* [161], serogroups B, C, Y and W cause approximately 90% of invasive meningococcal infections. Group A, however, is the only meningococcal serotype capable of causing of meningitis epidemics. Serogroups B and C express α-(2,8)- and α-(2,9)-linked polysialic acid, respectively. Alternating sequences of d-galactose or d-glucose and sialic acid are expressed by serogroups W and Y [162,163]. The serogroup A capsule is composed of α-(1,6)-linked *N*-acetyl-d-mannosamine-1-phosphate repeating units, partially acetylated at 3-OH (about 70%) and 4-OH (10–30%) [164]. First generation polysaccharide-based vaccines against *N. meningitidis* comprise the bivalent (groups A and C), the trivalent (groups A, C and W), and the tetravalent (groups A, C, Y and W) forms. Among second generation meningococcal glycoconjugate vaccines, three monovalent group C conjugate vaccines and one tetravalent meningococcal conjugate vaccine against groups A, C, Y and W are currently available. Of note, serogroup B (Men B) is not included in current formulations and remains a major cause of endemic meningitis in both developed and developing countries. The main obstacle for group B polysaccharides vaccine development is that the group B polysaccharide, composed of α-(2,8)-sialic acid polymers, is expressed in a number of human neurologic tissues since early fetal development. Men B CPS is therefore perceived as self-antigen by the innate immune system and it induces immune tolerance. Structural modification of this “self” antigen replacing the *N*-acetyl group of sialic acid units with an *N*-propanonyl group [165] induced high levels of bactericidal IgG antibodies without detection of autoantibodies [166]. However, its development has been suspended due to the poor performance of the vaccine in a limited human trial and to the high perceived risk of autoimmunity [61]. A new type of group B vaccine, Bexsero^®^ (GlaxoSmithKline) developed by conjugation of three recombinant surface antigens (PorA, NadA and fHbp) and outer membrane vesicles from group B strain NZ98/254, is now licensed in more than 35 countries worldwide, including the EU, Australia, Brazil, Canada, Chile, Uruguay and the USA [167,168,169]. More recently, a new anti-MenB vaccine based on two recombinant lipidated factor H binding protein (Trumenba^®^, Pfizer) has been licensed by FDA and approved for use in EU countries in 2017.

### 12.1. N. meningitidis Serogroup A (MenA)

**Figure 23:**
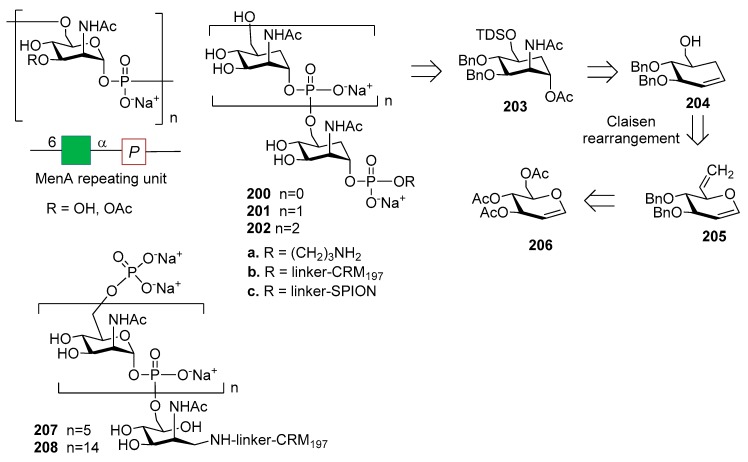
Men A CPS carbocyclic analogues reported in Reference [172].

*N. meningitidis* serogroup A is most often implicated in seasonal epidemic diseases, especially in sub-Saharan Africa and asian developing countries [170,171]. The MenA CPS structure consists of (1→6)-linked 2-acetamido-2-deoxy-α-d-mannopyranosyl phosphate repeating units, with about 70% of *O*-acetylation at 3-OH (Figure 23).

**Figure 24:**
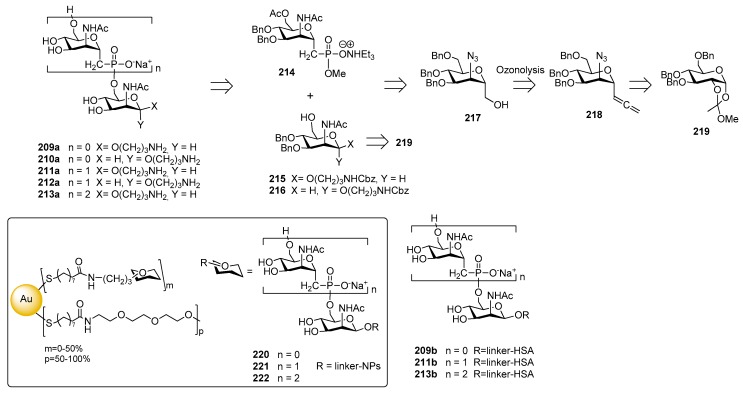
Retrosynthetic strategy for the synthesis of C-phosphono analogues of MenA CPS reported in [174] and multivalent presentation of the fragments [181].

The synthesis of MenA CPS fragments was reported in 2002 by Pozgay [173] and by Oscarson in 2005 [174], and upon conjugation to HSA the synthetic fragments were found to be immunogenic. MenA CPS, however, suffers from poor stability in water, due to the chemical lability of the phosphodiester linkages involving the anomeric position of each repeating unit. This issue stimulated the design of novel and hydrolytically stable analogues of MenA CPS repeating unit, like carbocyclic analogues (Figure 23) [172] and 1-C-phosphono analogues (Figure 24) [175,176], where a methylene group replaces the pyranose oxygen atom or the anomeric oxygen, respectively. The conformational behaviour of these analogues was investigated through DFT calculation and NMR spectroscopy [177], with a particular focus on the orientation of the phosphate or phosphonate aglycone and on the possibility of pyranose ring inversion [178]. The comparison between mimics and natural fragment showed the preservation of the ^4^C_1_ geometry in both classes of analogues. The synthesis of carbocyclic stabilized analogues of MenA CPS fragments was reported by the Lay group with the obtainment of monomer **200a**, dimer **201a** and trimer **202a** of carba-*N*-acetylmannosamine-1-*O*-phosphate. The formation of the phosphodiester bridges was achieved through the use of the H-phosphonate methodology [179], followed by functionalization with a phosphodiester-linked aminopropyl spacer to allow protein conjugation. Oligomer synthesis was achieved starting from carbasugar **203**, derived from compound **204**. Carbocycle formation was carried out by Claisen rearrangement of glucal **205**, in turn obtained from commercially available glucal **206** [172,177]. The inhibition abilities of the synthetic molecules were investigated by a competitive ELISA assay, showing that carba-disaccharide **201a** is recognized by a polyclonal anti-MenA serum with an affinity similar to a native MenA oligosaccharide with average polymerization degree of 3 [172]. The conjugation of carbocyclic analogues **200a**–**202a** to the protein carrier CRM_197_ gave glycoconjugates **200b**–**202b** (Figure 23) that were tested for immunogenicity [180]. MenA fragments, produced by mild acid hydrolysis of native MenA polysaccharide (average degree of polymerization from 6 to 15) and conjugated to CRM_197_ were used to compare the activity of **200b**–**202b**. Upon mice immunization, all glycoconjugates elicited antibodies that recognized the respective structures, although only conjugated trimer **202b** was able to induce specific anti-MenA IgG antibodies with detectable in vitro bactericidal activity. Compound **202b**, however, elicited antibodies to a lesser extent than hexamer and pentadecamer conjugated oligomers **207** and **208** obtained from hydrolysis of the native polysaccharide, suggesting that hydrolytically stable analogues of MenA CPS can be used for the development of vaccine and that conjugates with longer carbocyclic oligomers could further increase the induced immune response. In addition, a strategy for the multivalent presentation of carba analogues was developed [181] allowing conjugation of monomer **200a** and dimer **201a** to the metallic surface of superparamagnetic iron oxide nanoparticles (SPION) to generate **200c** and **201c** (Figure 23). SPIONs can act as multivalent carriers and as a contrast agent for magnetic resonance imaging (MRI) [182]. Functionalized SPIONs dispersed in aqueous media can aggregate into clusters inducing a reduction of *T2* [183] and this event can be monitored as a decrease in brightness of a *T2*-weighted MR image [183,184]. This property has been widely used for ligand detection in biological media [185]. SPIONs **200c** and **201c** were produced as approximately spherical nanoparticles, with a size dispersion of 13 ± 3 nm and an average particle coating of 320 unities per nanoparticle for **200c** and of 160 ligands per nanoparticle for **201c**, as determined by transmission electron microscopy (TEM). Both **200c** and **201c** were able to bind the polyclonal anti-MenA antibody, as evaluated by MRI analysis, exploiting the magnetic peculiarity of SPIONs.

The synthesis of C-phosphono analogues of *N. meningitidis* group A CPS oligomers was reported by the Oscarson [176] and Lay [186] groups. In particular, an improved strategy for the synthesis of monosaccharides **209a**–**210a** and phosphonoester-bridged fragments **211a**–**213a** was recently reported [175] starting from compound **214**, **215** and **216**. The introduction of the phosphonate moiety was accomplished on alcohol **217**, obtained from α-*C*-allenyl derivative **218** which, in turn, was prepared in six steps from orthoester intermediate **219** (Figure 24).

Competitive ELISA assay showed that monosaccharides **209a**–**210a** and synthetic fragments **211a**–**213a** containing the unnatural phosphonoester linkage were recognized by a human polyclonal anti-MenA serum [175]. The comparison with the inhibition of either MenA (positive control) or MenY (negative control) indicated that the chain lengths of the saccharide molecules is important for the efficacy, while the presence of the phosphonate residue (comparison between compounds **211a**–**213a** and glycosides **209a**–**210a**) and the orientation of the anomeric linker (comparison between compounds **211a** and **212a**) did not affect the affinity. Multivalent presentation on gold nanoparticles of monomer **209a**, dimer **211a** and trimer **213a** were obtained (GNPs **220**, **221** and **222** respectively, Figure 24) [187]. Interestingly, nanoparticles **220**, **221** and **222** showed a more than three order of magnitude higher binding affinity than their counterparts not bound to the gold cluster **209a**, **211a** and **213a**, at the same nominal concentration of saccharides. Fallarini et al. [188] used functionalized gold nanoparticles to test their ability to induce immune cell responses as a consequence of multivalency. In particular, monodisperse gold nanoparticles (2 and 5 nm) coated with mono- and disaccharides (**220** and **221**) were synthesized. Conjugation to gold nanoparticles conferred to the saccharides the ability to activate macrophages and this property is dependent on the size of the nanoparticles, with 5 nm nanoparticles giving comparable results to those obtained with the polysaccharide bacterium capsule (MenA) used as a natural antigen. Activation of macrophages occurred, independently of the saccharide oligomerization (or charge) on the nanoparticle surface. However, only nanoparticles **220**, exposing a phosphonodisaccharide-functionalized monolayer, induced T cells proliferation and the increase of released interleukin-2 levels, the latter being a typical marker of T cell activation. Recently, HSA conjugates **209b**, **211b** and **213b** [189] were shown to induce both T cell proliferation and interleukin-2 release in vitro, and to stimulate moderate specific IgG antibody production in vivo. All HSA-conjugated compounds **209b**, **211b** and **213b** induced T cell proliferation (40% of proliferation at 102 μM), whereas only phosphonodisaccharide **211a** was effective (28% of proliferation at 102 μM) among the unconjugated forms, showing the unusual behavior of triggering T cell proliferation in vitro and causing interleukin-2 release.

### 12.2. N. meningitidis Serogroup C (MenC)

**Figure 25:**
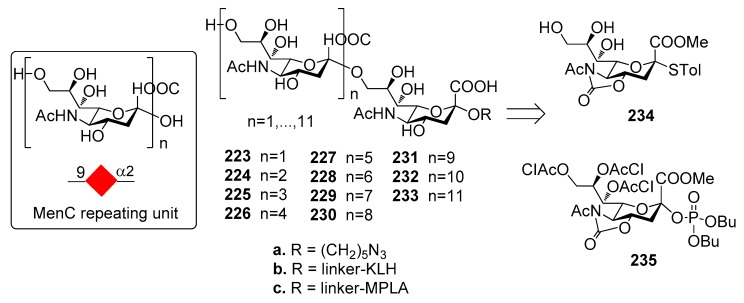
Repeating unit of MenC CPS and synthetic fragments reported in [191,192].

*N. meningitidis* group C CSP is a α-(2,9)-polysialic acid with sporadic 7/8-*O*-acetylation (Figure 25). Non-acetylated fragments have been shown to be immunogenic and to elicit an immune response that is effective in recognizing and killing the bacterium [190]. A series of non-acetylated α-2,9-oligosialic acids of different length **223a**–**233a** were prepared by a convergent synthetic route employing 5*N*,4*O*-oxazolidinone-protected phosphate-based building blocks **234** and acceptor **235** [191]. The dodecamer was synthesized with a [4+8] strategy that allowed to retain the α-selectivity even when the size of donor and acceptor increased.

In a further study, di-, tri-, tetra-, and penta-sialic acids **223a**–**226a** were coupled with KLH carrier protein and studied for immunogenicity [192]. The glycoconjugates elicited robust T cell-mediated immunity in mice, in particular the immunogenicity of the tested oligo sialic acids increased in the order tri(**224b**) > di(**223b**) > tetra(**225b**) > penta(**226b**). The antibodies elicited were tested for efficacy and specificity, verifying if they could recognize and target group C *N. meningitidis*. All of the antisera obtained with the oligosaccharide-conjugates **223b**–**226b** had strong binding to the *N. meningitidis* cell and no significant binding to cells not expressing α-2,9-poly and oligosialic acids (sialoglycans sTn, GM3, GM2, α-2,8-linked polysialic). The α-2,9-trisialic acid was identified as a promising antigen for developing glycoconjugate vaccines against group C *Neisseria meningitidis*. Recently, α-2,9-oligosialic acid fragments (di-, tri-, tetra-, and penta, **223a**–**226a**) were conjugated with monophosphoryl lipid A (MPLA), a known immune potentiator (molecule with adjuvant activity) [193]. Immunological studies of the conjugates **223c**–**226c** (Figure 25) in mice revealed that they elicited robust immune responses, mainly IgG2b and IgG2c, consistent with T cell dependent immunities. In particular, the immune response was comparable to the corresponding KLH-conjugates **223b**–**226b** plus adjuvant. The immunogenicity of oligosialic acids decreased with elongated sugar chain, although all tested MPLA conjugates exhibited strong immune responses. The dimer **223c**, actually, induced the highest titers of antigen-specific total and IgG2b antibodies, but some of the produced antibodies did not bind to oligosialic acids or bacterial CPS. In contrast, the tri- and tetra-sialic acid−MPLA conjugates **224c**–**225c**, were fully effective showing the highest binding to bacterial cells and were identified as promising vaccine candidates worthy of further investigation.

### 12.3. N. meningitidis Serogroup W (MenW)

**Figure 26:**
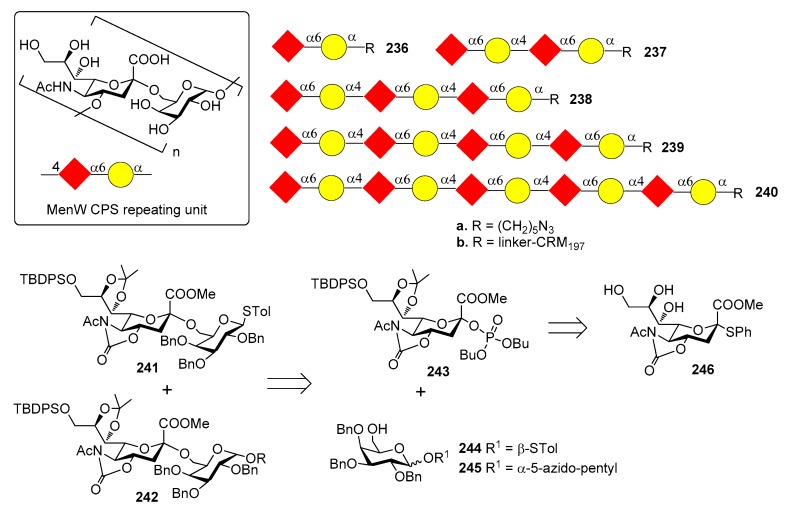
Repeating unit of MenW CPS and synthetic strategy used the construction of MenW CPS fragments [194].

*N. meningitidis* serogroup W CPS consists of a glycan repeating unit of [→6)-α-d-Gal*p*-(1→4)-α-d-Neu*p*5Ac(7/9OAc)-(2→] (Figure 26). The synthesis of MenW CPS oligosaccharides of various lengths was performed by Wu group in 2013 [194], with the aim of studying the relationship between oligosaccharide length and immunogenicity.

Oligomers up to decasaccharides **236a**–**240a** were obtained starting from protected disaccharides **241** and **242**, bearing a *N*-acetyl-5-*N*,4-*O*-oxazolidinone (Figure 26), in turn synthesized from sialyl phosphate donor **243**, functionalized with an oxazolidinone and a phosphate leaving group to increase the α-selectivity, and galactosides **244** and **245**. Compound **243** was prepared from compound **246**. Oligosaccharide elongation was accomplished by iterative glycosylation and deprotections, using disaccharide **241** for the [2+n] glycosylation reactions. After conjugation with CRM_197_, immunization of mice and glycan array analysis of produced antisera showed that antibodies induced by conjugated disaccharide **236b** recognized only the disaccharide itself and did not cross react with longer oligomers. In contrast, antibodies induced by glycoconjugates **237b**, **238b**, **239b** and **240b** all recognized tetra- to decasaccharides, but not disaccharides. A serum bactericidal assay was used to study the bactericidal activity of the antibodies and showed that **236b** did not induce antibodies with bactericidal activity. On the contrary, longer oligomers could and in particular, the tetramer **239b** elicited antibodies with the highest bactericidal effect. Taken together, these data suggested that the tetrasaccharide is the minimum saccharide length required to induce bactericidal antibodies.

### 12.4. N. meningitidis Serogroup X (MenX)

**Figure 27:**
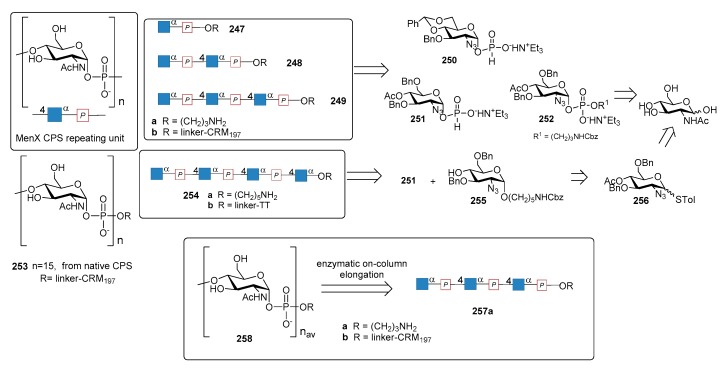
Repeating unit of MenX CPS and synthetic fragments reported in [200,201,202].

Serotype X of *N. meningitidis* (MenX) emerged as a substantial threat to public health, especially in the “meningitis belt” area, after the introduction of MenA vaccine (MenAfriVac) and other conjugate vaccines (consisting of MenA, C, Y and W serotypes) that do not provide coverage for MenX serotype [195,196]. As a consequence, there is the need to extend the protection of current vaccines, developing more comprehensive conjugate vaccines [197]. The MenX CPS is a homopolymer of 2-acetamido-2-deoxy-α-d-glucopyranosyl phosphate moiety (Figure 27) [198,199]. 

In 2013 Morelli et al. reported the synthesis of monomer **247a**, dimer **248a** and trimer **249a** of *N. meningitidis* X CPS [200], starting from intermediates **250**–**252**, in turn derived from d-GlcNAc.

In 2014 an improvement of the synthesis of MenX fragments, their conjugation to CRM_197_ and the immunological evaluation of the resulting conjugates **247b**–**249b** was reported [203]. Upon mice immunization, the conjugated trimer (**249b**) was found as the minimal fragment possessing immunogenic activity, although significantly lower than pentadecasaccharide-conjugate **253** obtained from the native polymer and used in the same study. This finding suggests that oligomers longer than three repeating units were possibly needed to mimic the activity of the native polysaccharide. The following year, Harale et al. reported an alternative synthesis of the tetrameric fragment of MenX CPS **254a**, lacking the phosphate at the downstream end, starting from intermediates **251** and **225** in turn synthesized from monosaccharide **256** (Figure 27) [201]. Competitive ELISA experiment, using MenX bacterial CPS as a control, gave a concentration-dependent inhibition of anti-MenX antibodies from both compound **254a** (unconjugated synthetic MenX tetramer) and tetramer-TT conjugate **254b**. Lower inhibition for unconjugated fragment **254a** (up to 68% inhibition) was observed compared to its conjugate form **254b** (up to 89% inhibition) at all antigen concentrations tested. Also, bacterial MenX CPS showed higher inhibition than synthetic compounds at all respective concentrations used [201]. Very recently, an alternative strategy for MenX oligomer synthesis (Figure 27) was developed [202], based on an enzyme-catalyzed one-pot elongation of synthetic trimer **257a**. Oligomers with predefined average length (compound **258a** for general formula of avDP = 12) were synthesized and conjugated to CRM_197_. Mice immunized with **258b** elicited functional antibodies comparable to controls immunized with the current MenX glycoconjugates prepared from the natural CPS or from its fragments enzymatically produced.

## 13. *Mycobacterium tuberculosis*

**Figure 28:**
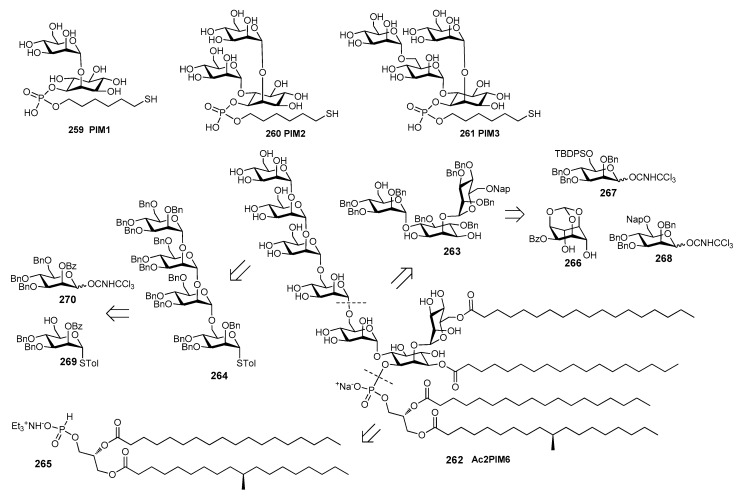
Phosphatidylinositol mannosides (PIMs) and retrosynthetic route of tetraacylated phosphatidylinositol hexamannoside (Ac_2_PIM_6_) reported in [223].

Tuberculosis (TB), caused by *Mycobacterium tuberculosis* (Mtb), remains one of the leading causes of death in the world. In 2016, some 1.7 million people died from the disease, with 64% cases of the total occurring in India, Indonesia, China, Philippines, Pakistan, Nigeria, and South Africa [204]. Moreover, it is estimated that one-third of the human population is latently infected with *M. tuberculosis* and is highly vulnerable if immunocompromised (in 2016, 40% of HIV deaths were due to TB). The mycobacterial cell wall is a highly complex structure largely composed of carbohydrates and lipids. Major components are lipidated polysaccharides, like the mycolyl−arabinogalactan complex, essentially composed of galactofuranose (Gal*f*) and arabinofuranose (Ara*f*) [205]. In recent years, numerous efforts have been done to develop inhibitors of UDP-galactopyranose mutase (UGM), an enzyme essential for the growth and survival of this mycobacterium [206,207,208,209,210,211,212]. Among the vital cell envelope components, phosphatidylinositol mannosides (PIMs) and their corresponding hyper-mannosylated derivatives (lipomannans and lipoarabinomannans) are noncovalently anchored to the plasma membrane and the outer capsule via their lipid chains. PIMs have a crucial role in the intracellular life of the bacterium, by binding to macrophages, to Toll-like receptors and C-type lectins [213,214], expressed on antigen presenting cell surfaces. PIMs also activate natural killer T cells for the production of interferon-γ [215]. Structurally, PIMs consist of a myo-inositol moiety linked with a diacylated glycerophospholipid unit and two α-mannosylation sites at O2 and O6 (Figure 28, compounds **259**–**261**). When additional lipid chains are linked to the mannosyl-and myo-inositol-moieties, triacylated PIMs (AcPIMs) and tetraacylated PIMs (Ac2PIMs) are formed. Higher PIMs (for example, AcnPIM3 AcnPIM6) are formed by elongation at the mannose residue. In the course of the past few years, several synthesis of these structures have been reported [216,217,218,219,220,221]. In addition, heterocyclic analogues of PIMs in which the inositol ring is replaced by a piperidine or a tetrahydropyran moiety have been prepared and shown to retain the biological activity of the parent PIM structures [222].

Recently, the synthesis of a tetraacylated phosphatidylinositol hexamannoside (Ac_2_PIM_6_, **262**) and its immunological evaluation have been reported [223]. Oligomer **262** was synthesized starting from pseudotrisaccharide **263**, tetramannoside donor **264** and hydrogen phosphonate **265** (Figure 28). Compounds **263** and **264** were in turn obtained from monosaccharides **266**, **267**, **268** and **269**, **270**, respectively through a [1+1] strategy. The immunological evaluation was performed by observing the induction of antigen-specific antibodies in mice immunized with ovalbumin or tetanus toxoid adjuvanted with compound **262**. The adjuvant effects of Alum or various PIMs isolated from *M. tuberculosis* strain H37Rv (iPIM1,2 and iPIM6) were also examined in parallel for comparison. Mice exposed to the synthesized Ac_2_PIM6 **262** exhibited increased production of interleukin-4 and interferon-γ, suggesting proper activation of the innate immune system. Interestingly, **262** induced an approximately two to four-fold increase in the level of antigen specific antibodies, similarly to bacteria-derived PIMs and slightly lower than Alum.

**Figure 29:**
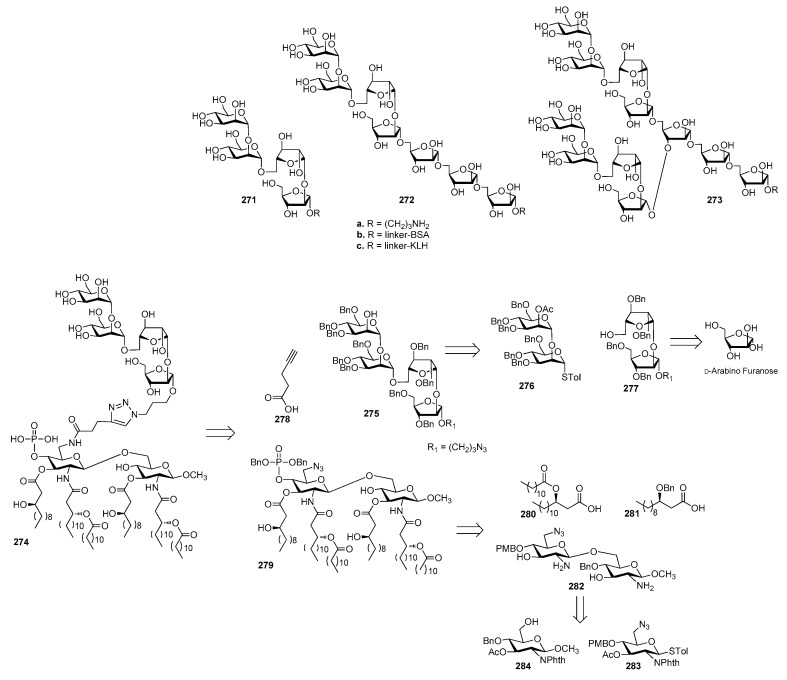
LAM oligosaccharides reported in Reference [83] and retrosynthetic route to LAM tetrasaccharide coupled to a monophosphoryl lipid A (MPLA), as reported in [226].

Among *M. tuberculosis* cell wall polysaccharide and lipid components, some complex structures of arabinogalactan and lipoarabinomannan (LAM) have been synthesized in recent years [224,225]. In particular, Wang et al. [83] synthesized LAM oligosaccharides **271a**–**273a** (Figure 29), that were conjugated to BSA (**271b**–**273b**) and KLH (**271c**–**273c**) and evaluated with immunological studies.

LAM tetra-, hepta-, and undecasaccharides **271a**–**273a**, containing the α-1,5-, α-1,3-, and β-1,2-linked arabinan domain with the 5-OH of the upstream residues capped with the α-1,2-linked dimannose motif, were synthesized in good overall yields from d-arabinose in 10, 15, and 14 linear steps, respectively [83]. KLH conjugates **271c**–**273c** and free oligosaccharides **271a**–**273a** were injected in mice and the obtained antisera were analyzed by ELISA using BSA conjugates **271b**–**273b** as capture antigens. Antisera derived from mice immunized with oligosaccharides **271a**–**273a** did not contain carbohydrate antigen-specific antibodies, while all KLH conjugates **271c**–**273c** elicited antigen-specific immune responses in mice. In particular, antibody titers induced by **271c** and **273c** were slightly higher than those induced by **272c**. In a further work [226], the LAM tetrasaccharide was coupled to a monophosphoryl lipid A (MPLA) derivative generating a MPLA-based synthetic glycoconjugate **274** (Figure 29). In **274**, the carbohydrate antigen was attached to the MPLA C-6′-position via an amide bond. Retrosynthetic analysis of **274** gives LAM tetrasaccharide **275**, conveniently synthesized with a [2+2] strategy from **276** and **277** as reported before by the same authors [83], linker **278**, and MPLA derivative **279**. In turn, **279** was synthesized from fatty acids **280** and **281** and disaccharide **282**, assembled from glycosyl donor **283** and acceptor **284**. Immunological activity of conjugate **274** was evaluated in mice, affording robust IgG antibody responses. Since intraperitoneal injection elicited responses significantly stronger than those from subcutaneous injection, it was hypothesized that MPLA conjugates may stimulate B1 lymphocytes in the intrapleural and peritoneal cavities. These results revealed also the self-adjuvant properties of MPLA conjugates, paving the way to further investigation of these compounds as antituberculosis vaccine candidates.

## 14. Fungal Infections

**Figure 30:**
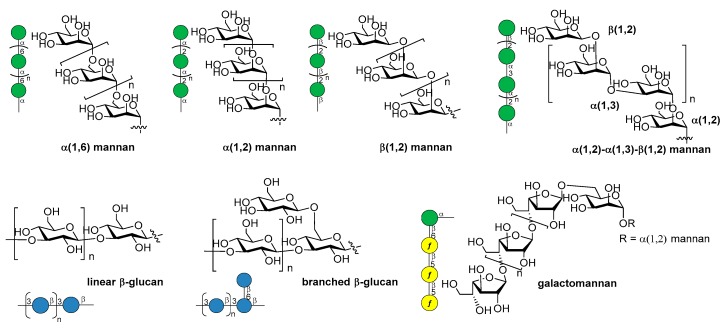
Examples of mannan, β-glucan and galactomannan components of fungal cell walls.

Fungal infections can occur in healthy people, although immunosuppressed or immunocompromised patients are the major risk group for invasive fungal infections, as well as patients who use antibiotics able to modify the human microbiota [227]. Antifungal drugs are commercially available but they are limited compared to antibacterial drugs. In recent years, several strategies have been developed for the identification of new anti-fungal compounds, including components of plants, animals and microorganism [227]. In particular, vaccination offers promising alternative solutions for the treatment of fungal infections that are resistant to antibiotics. Recognition of fungi by the innate immune system depends on several Pathogen-Associated Molecular Patterns (PAMPs) in the fungal cell wall [228]. Specific receptors, exposed on antigen presenting cells surface, are involved in the recognition of polysaccharide cell wall components, like the mannose receptor (MR) and DC-SIGN for recognition of branched *N*-linked mannan [229], Toll-like receptor 4 (TLR4) for recognition of linear *O*-linked mannan [230], galectin 3 for β-mannosides, complement receptor 3 (CR3) for β-(1,6)-glucan, and dectin 1 and TLR2 for β-glucan and phospholipomannan [231]. Mannans consist of differently linked oligomannoside, and phospholipomannans are composed of phospholipids and mannans. The common linkages between mannose units in fungal mannans are α-(1,6), α-(1,2)-, α-(1,3) and β-(1,2), as shown in Figure 30. β-glucans are composed of β-d-glucose with β-(1,3) linkages and sporadic β-(1,6) branched points. Galactomannans consist of structurally diverse heteropolysaccharides composed of a poly-d-mannose backbone linked to galactofuranoside (Gal*f*) units (Figure 30). Of note, the galactomannan of *Aspergillus fumigatus* is a specific carbohydrate antigen used for clinical detection of fungal infection.

In the course of the past few years, several synthetic strategies [232,233,234] have been developed to prepare these oligosaccharides expressed on the cell wall of various fungi, like *Candida albicans* and *Apergillus fumigatus*, as detailed in Section 14.1 and Section 14.2.

### 14.1. Candida albicans

**Figure 31:**
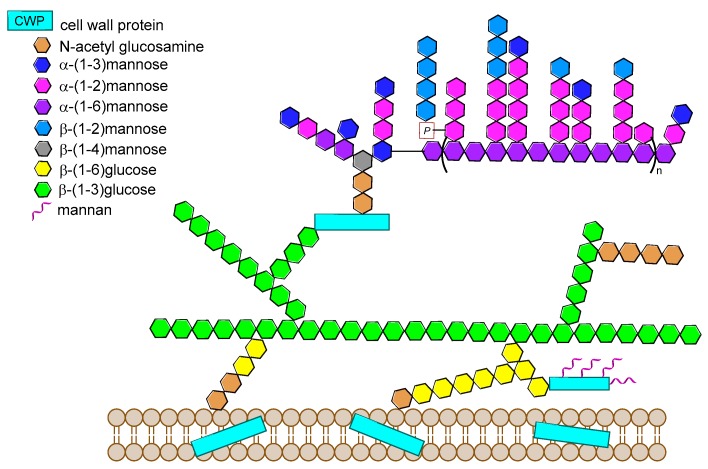
Major components of *C. Albicans* cell wall: β-(1,3)-glucan and chitin (poly-β-(1,4)-*N*-acetylglucosamine) are the main components of inner cell wall. The outer layer is enriched with polymannans.

The yeast *Candida albicans* is an opportunistic pathogenic microorganism, found in the normal microflora, skin and the mucosal surfaces of most healthy individuals. However, it is able to cause severe infections in immunocompromised individuals and patients undergoing immunosuppressive therapy [235]. The three major glycans expressed on the cell wall of *C. albicans* are phosphomannan-based glycoproteins in the outermost part of the cell wall and β-glucans and chitin, especially at the level of the bud scars (Figure 31) [228].

**Figure 32:**
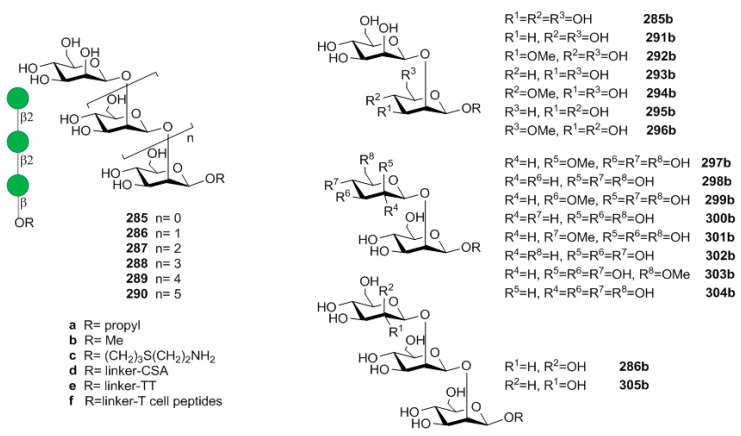
Oligomannans and glycoconjugates synthesized by the Bundle group [236].

Being part of the phosphomannan glycoproteins, the acid-labile β-mannan is thought to be a major epitope of all *C. Albicans* serotypes and thus represents an ideal target for vaccine development. On this subject, an extensive work has been carried out by the Bundle group to develop a conjugate vaccine against *C. albicans*, starting from binding studies of β-mannans with two monoclonal protective antibodies, the mAb C3.1 and its immunoglobulin M (IgM) counterpart B6.1 [236]. By means of STD-NMR, computational analysis [237] and hydroxyl group replacement [238], the identification of key recognition elements was used for the development of antigens that elicit polyclonal protective antibodies, a process referred to as “reverse engineering” [236]. Initially, a set of β-(1-2)-mannan oligosaccharide propyl glycosides **285a**–**290a** (Figure 32) ranging in size from di-to heptasaccharides were evaluated against mAbs C3.1 and B6.1. Interestingly, di and trisaccharides **285a** and **286a** had maximum binding capacity, while larger oligosaccharides were bound progressively more weakly [239]. This unusual pattern of inhibition was consistent with a binding site that could accommodate the trisaccharide, even though the primary polar contacts are located within the disaccharide. The synthesis of complementary mono-deoxy and mono-*O*-methyl analogues of dimers **285b**, **291b**–**304b** and trimers **286b** and **305b** (Figure 32) led to epitope mapping of anti-*Candida albicans* antibodies [240]. The strategy for the construction of these β-mannosides is based on the formation of β-glucosidic linkages, followed by epimerization at C-2 via an oxidation−reduction sequence.

Dimer **285c** and trimer **286c** (R = (CH_2_)_3_S(CH_2_)_2_NH_2_) were conjugated to chicken serum albumin (CSA) and the resulting glycoconjugates **285d** and **286d** (Figure 32) were able to generate highly specific IgG Abs titers in mice and rabbit [241]. The trisaccharide conjugated to CSA (**286d**) raised protective antibodies in rabbits [242]. Serum from rabbits immunized by **286d** contained antibodies that stained *C. albicans* cells in fluorescent labeling studies more intensely than antibodies from rabbits immunized with **285d** [105,241]. Both glycoconjugates **285d** and **286d** reduced *C. albicans* counts in vital organs but they were insufficient to provide 100% protection. In a further work, trisaccharide–TT conjugate **286e** was found to be a stronger immunogen in rabbits, but it was poorly immunogenic in mice [241]. However, when the same trisaccharide was conjugated to different T cell peptides, found in cell wall proteins expressed during pathogenesis of human candidiasis, the resulting glycopeptides (**286f**) elicited a peptide- and carbohydrate-specific response that gave protection against challenge by *C. albicans* infection in mouse models [243]. All glycoconjugates showed immunogenicity with higher Abs titers compared to unconjugated **286c**. When hyphal wall protein-1 (Hwp1), fructose-bisphosphate aldolase (Fba) and methyltetrahydropteroyltriglutamate (Met6) were used as T cell peptides for conjugation, the survival rate in mice challenge experiments was 80–100% against 40–80% survival with other glycopeptides.

**Figure 33:**
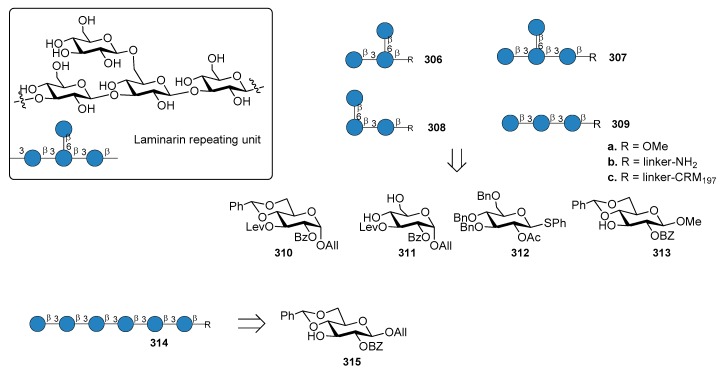
Repeating unit of Lamarin (Lam) and Lam fragments reported in [247].

The *C. albicans* cell wall consists of approximately 60% β-glucan. Although initially thought to be hidden underneath the mannoprotein layer, recent evidence suggest that β-glucans are exposed on the cell surface, possibly restricted to specific regions, such as bud scars [228]. β-glucans have also been investigated for their binding capacity to dectin-1 [244,245], a dendritic cell receptor that mediates phagocytosis and mediator production during inflammation caused by fungal pathogens [231]. Among β-glucans, Laminarin (Lam) is a polysaccharide extracted from *Laminaria digitate* plant. In 2010, Bromuro et al. [246] investigated the potential of laminarin as antigen for *C. albicans*, aimed at mediating antifungal protection. The authors showed that a synthetic linear structure composed of pentadecamer of Lam β-(1-3) repeating units (15mer) conjugated to CRM_197_ conferred protection against *C. albicans*. In the same study, a synthetic β-(1-6) branched 17mer conjugated to CRM_197_ was immunogenic but not protective [246], suggesting that the linear β-(1-3) fragment contains the protective epitope. To further investigate this aspect, Adamo et al. [247] reported the synthesis of short linear and β-(1-6) branched Lam fragments **306a**–**309a** (Figure 33) and their immunological evaluation.

Compounds **306a**–**315a** were synthesized starting from common building blocks **310**, **311**, **312** and **313** (Figure 33). Preliminary ELISA test with fragments **306a**–**309a** showed that compound **309a** was the best inhibitor of the binding between Lam and anti-Lam Abs elicited in mice by Lam-CRM_197_ conjugate, confirming the hypothesis that the linear β-(1-3) fragments contain the dominant epitope. Hexasaccharide **314b**, prepared from building block **315** (Figure 33), was studied in order to identify the protective epitope. ELISA assays on compounds **306a**–**309a** and **314b** showed that hexasaccharide **314b** provided 95% of inhibition at 4 mM concentration, while trisaccharide **309a** and tetrasaccharide **307a** provided 85% and 73% of inhibition at the same concentration, respectively. Based on these results, compound **314b** was conjugated to CRM_197_ and employed for mice immunization. Sera analysis showed that glycoconjugate **314c** was able to induce specific anti-Lam IgG Abs titers significantly higher and homogeneous compared to Lam-CRM_197_ conjugate. This result suggested that the linear hexasaccharide β-(1-3) glucan **314b** is long enough to cover the dominant epitope of *C. albicans.*

**Figure 34:**
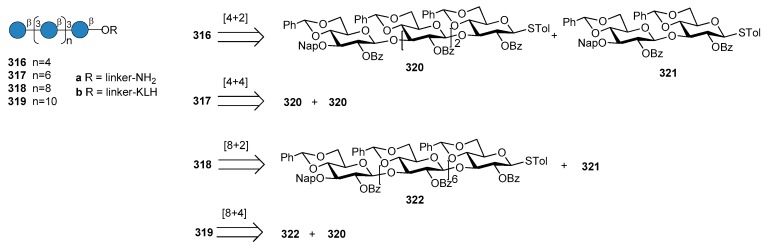
Hexa-, octa-, deca- and dodecasaccharide of β-glucan reported in Reference [248].

In 2015, Liao et al. [248] reported the synthesis of longer β-(1-3) glucan chains such as fragments **316a**–**319a** (Figure 34). Hexasaccharide **316a** was assembled with a [4+2] glycosylation reaction from donor **320** and compound **321** after cleavage of 2-Naphthylmethyl ether (Nap) group. Longer oligomers **317a**, **318a** and **319a** were obtained via [4+4] (from **320**), [8+2] (from **322** and **321**) and [8+4] (from **322** and **320**) glycosylation strategies, respectively (Figure 34). Oligomers **316a**–**319a** were conjugated to KLH and KLH conjugates (**316b**–**319b**) were injected in mice, raising high IgG1 titers with significant statistical difference between hexasaccharide **316b** and longer oligomers **317b**–**319b**. Octasaccharide **317b** resulted the most immunogenic and protective compared to decasaccharide **318b** and dodecasaccharide **319b**. This result encouraged the authors to perform fungal challenge experiment in mice with glycoconjugate **317b** using *C. albicans* fungus strain SC5314. In this experiment, 11 mice were immunized with **317b** and, after challenge, 4 mice (about 34%) were unaffected, suggesting their protection from *C. albicans*.

**Figure 35:**
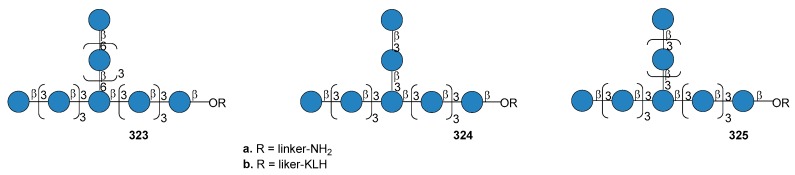
Structure of fragments **285a**–**287a** and their KLH conjugates [249].

More recently, Liao et al. [249] reported similar immunological studies of fragments **323a**–**325a** containing the dominant linear octasaccharide and β-(1-6) and β-(1-3) branches with different chain length (Figure 35). KLH conjugates **323b** and **324b** were more immunogenic than **325b** but the majority of elicited Abs were against the common β-glucan motif. In addition, conjugates **323b** and **325b** confer similar long-term protection against *C. albicans* infection (survival rate about 37%, 30 days after challenge for both conjugates). These data confirmed the hypothesis that linear β-(1-3) glucans of 6–8 units without branches can cover the dominant epitope of *C. albicans*.

### 14.2. Aspergillus fumigatus

**Figure 36:**
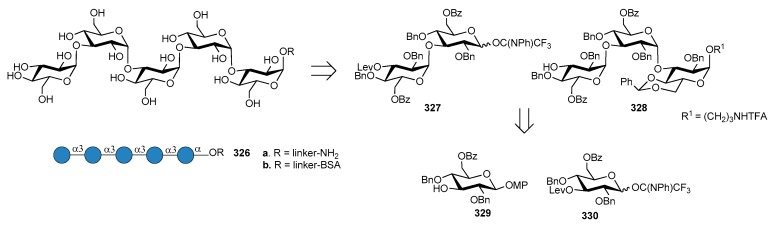
Retrosynthetic analysis of pentasaccharide **326a**, as reported in [250].

*Aspergillus fumigatus* causes severe and usually fatal invasive aspergillosis infections in immunosuppressed hospitalized patients. More than 90% of the cell wall of *A. fumigatus* comprises polysaccharides, among which α-(1,3)-glucan is present in percentages variable from 20 to 40%. This virulence factor suffers of poor solubility in water, making difficult the study of its immunological properties. The use of shorter synthetic fragments could overcome the solubility issue. Indeed, Komarova et al. [250] reported the synthesis of pentasaccharide fragment **326a** (Figure 36), the BSA-glycoconjugate (**326b**) and its evaluation as a vaccine candidate. Pentasaccharide **326a** was prepared via a [3+2] glycosylation between donor **327** and acceptor **328**. These protected fragments were in turn synthesized from monomers **329** and **330**. The use of benzoyl group at 6-OH as remote partecipating group during glycosylation reactions facilitated the formation of α-linkages. Glycoconjugate **326b** was immunized in mice, eliciting highly specific and protective polyclonal antibodies.

**Figure 37:**
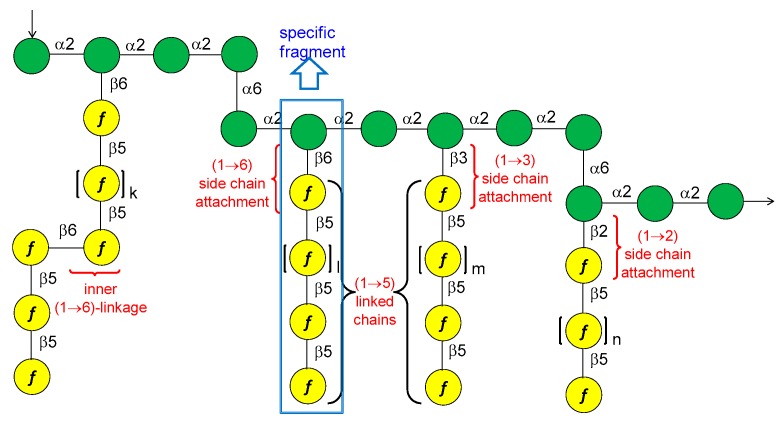
*A. fumigatus* galactomannan structure, underlining the presence of β-(1,6)-linked Gal*f* residues in addition to the β-(1,5)-linked Gal*f* residues, as reported in [252].

Galactomannan is a specific polysaccharide produced by *A. fumigatus* composed of a linear mannan core partially branched with β-(1,5)-galactofuranoside (Gal*f*) units via β-(1,6) or β-(1,3) linkages (Figure 37) [251]. The length of β-(1,5)-Gal*f* branches differs among different cultures, making difficult the identification of a common repeating unit for galactomannans. Recently, Kudoh et al. [252] demonstrated the presence of significant structural differences in both the O-linked and N-linked oligosaccharide of *A. fumigatus* galactomannans, depending on growth conditions in different culture media. Moreover, the authors revealed new structural elements of *A. fumigatus* galactomannan, the like the presence of β-(1,6)-linked Gal*f* residues in addition to the β-(1,5)-linked Gal*f* residues (Figure 37).

The galactofuranosides (Gal*f*) are not presents in mammalian cells and they are therefore used for diagnosis of human infections. In addition, they can be advantageously used as antigen candidates for an anti *A. fumigatus* vaccine.

**Figure 38:**
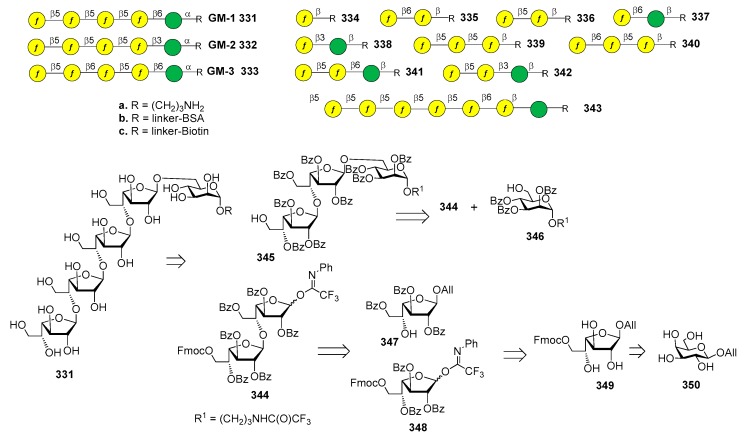
Synthetic fragment of *A. fumigatus* galactomannans reported in [254,255].

Recently, the Nifantiev group reported the synthesis of pentasaccharide fragments of the galactomannan containing the β-(1,5)-linked galactofuranoside chain attached to O-3 or O-6 of a mannopyranoside residue (GM-1 **331a**, GM-2 **332a** and GM-3 **333a**) [253] and fragments **334a**–**343a** (Figure 38) [254]. In particular, pentasaccharide **331a** was achieved via [2+3] glycosylation between disaccharide donor **344** and trisaccharide acceptor **345** (Figure 38), in turn prepared from donor **344** and mannosyl acceptor **346**. Disaccharide **344** was synthesized from monosaccharides **347** and **348**, both derived from allyl galactoside **350** through furanoside **349** (Figure 38).

The authors showed [255] that two mAbs (7B8 and 8G4), recognizing galactomannan in *A. fumigatus*, were elicited in mice after immunization with glycoconjugate **331b**. Glycoarray analysis performed with synthetic biotinylated oligosaccharides **331c**–**343c** and mAbs 7B8 and 8G4 showed high affinity towards pentasaccharide **331c** and heptasaccharide **343c** while no recognition was observed for mono- and disaccharides **334c**–**338c**. Finally, confocal microscopy was used to evaluate the binding between the two mAbs and *A. fumigatus* and a series of other fungi and bacteria. mAbs 7B8 and 8G4 were selective for *A. fumigatus*, suggesting that they can be used as reagents for immune diagnostics. Recent advances in galactofuranoside synthesis [205,232,233] should facilitate the access to galactomannan fragments, thus opening the way to anti *A. fumigatus* vaccines based on synthetic Gal*f* oligomers.

## 15. Conclusions

Pathogen surface glycans are promising vaccine targets due to their crucial role in adhesion to host tissues. Firstly, pathogen polysaccharides act as a protection barrier either by delaying the host’s immune response or by mimicking the host self-glycans. They are therefore essential for the survival of microorganisms in the blood and play a major role in their virulence. Secondly, most glycans are recognized by antigen presenting cells and triggers the innate immune response, starting the inflammatory process and eventually the antigen-specific adaptive immunity. Based on these considerations, the highly conserved polysaccharides (PS) exposed on pathogens cells surface are important virulence factors and have been used as antigens for vaccines development in a range of infectious diseases, including meningitis, pneumonia, otitis media, sepsis, infectious diarrheas, etc... The first-generation polysaccharide vaccines made of purified T cell-independent polysaccharide antigens have limited efficacy in infants, in the elderly and immunocompromised individuals and, in general, they don’t result in B cell-mediated immunological memory. The second-generation polysaccharide vaccines consist of native PS, produced and purified from natural sources and chemically conjugated to immunogenic carrier proteins able to elicit a T cell-dependent immune response (glycoconjugate vaccines). The purified native PS displays heterogeneity of chain length and may contain copurified endotoxin or other polysaccharides, introducing complications into the manufacturing process. Recent advances in carbohydrate chemistry has opened the way to a third-generation of polysaccharide vaccines, based on the use of synthetic oligosaccharides conjugated to carrier protein. The breakthrough in synthetic vaccines was carried out for *H. influenzae type* b in 2004, when the Vérez Bencomo research team in Cuba synthesized the first synthetic conjugate vaccine currently available under the trade name Quimi-Hib [9,10,11,12,13,14,15,16,17,18,19,20,21,22,23,24,25,26,27,28,29,30,31,32,33,34,35,36,37,38,39,40,41,42,43,44,45,46,47,48,49,50,51,52,53,54,55,56,57,58,59,60,61,62]. This approach is based on the hypothesis that protein conjugates of synthetic oligosaccharide fragments, smaller than the native PS, can elicit PS-specific and protective antibodies. The main challenge for the development of effective glycoconjugate vaccines is the determination of the carbohydrate antigen chain length and the sequence of the minimal protective immunogenic fragment of the antigen, a process often referred to as *epitope mapping*. The optimal carbohydrate antigen chain length needed for inclusion in a glycoconjugate vaccine, for example, cannot be predicted a priori for any given bacterial species. Despite the old paradigm established by Kabat [256], stating that immunogenic glycan epitopes usually comprise structures not longer than six-eight sugar units, the effect of the carbohydrate chain length on the immunogenicity of the corresponding protein conjugate is strictly case-dependent. There are many reported cases where both long chain glycans and oligosaccharides as short as tetrasaccharides have been shown to possess the minimal structural requirements for raising protective immunity, and several examples are illustrated throughout this review. In this regard, synthetic chemistry is a formidable tool to define the role of this crucial parameter, and more generally speaking of each structural variable affecting the immunogenicity and efficacy of vaccine candidates. Over the last years, much progress has been made. The rational design of carbohydrate antigens and the use of increasingly sophisticated synthetic strategies has made the preparation of even highly complex microbial glycans possible. In addition, the application of modern site-selective protein conjugation strategies [6,257,258] (a subject not addressed in the present review), has enabled the preparation of chemically defined glycoconjugate vaccine candidates featured by robust structure-immunogenicity relationship, which are expected to display improved safety and efficacy profiles [259]. These new achievements have certainly opened up new perspectives and hope for the prevention of severe bacterial and fungal infections still affecting the pediatric population worldwide. We truly wish that these promising results translate into new and efficient vaccines in the next few years.
